# Study on cocoonase, sericin, and degumming of silk cocoon: computational and experimental

**DOI:** 10.1186/s43141-021-00125-2

**Published:** 2021-02-16

**Authors:** Preeti Anand, Jay Prakash Pandey, Dev Mani Pandey

**Affiliations:** 1 Department of Bio-Engineering, Birla Institute of Technology, Mesra, Ranchi, Jharkhand 835215 India; 2 Central Tasar Research and Training Institute, Piska- nagri, Jharkhand Ranchi, India

**Keywords:** *Antheraea mylitta*, Cocoonase, Degumming, OCT, SEM, Sericin, Tasar silkworm

## Abstract

**Background:**

Cocoonase is a proteolytic enzyme that helps in dissolving the silk cocoon shell and exit of silk moth. Chemicals like anhydrous Na_2_CO_3_, Marseille soap, soda, ethylene diamine and tartaric acid-based degumming of silk cocoon shell have been in practice. During this process, solubility of sericin protein increased resulting in the release of sericin from the fibroin protein of the silk. However, this process diminishes natural color and softness of the silk. Cocoonase enzyme digests the sericin protein of silk at the anterior portion of the cocoon without disturbing the silk fibroin. However, no thorough characterization of cocoonase and sericin protein as well as imaging analysis of chemical- and enzyme-treated silk sheets has been carried out so far. Therefore, present study aimed for detailed characterization of cocoonase and sericin proteins, phylogenetic analysis, secondary and tertiary structure prediction, and computational validation as well as their interaction with other proteins. Further, identification of tasar silkworm (*Antheraea mylitta*) pupa stage for cocoonase collection, its purification and effect on silk sheet degumming, scanning electron microscope (SEM)-based comparison of chemical- and enzyme-treated cocoon sheets, and its optical coherence tomography (OCT)-based imaging analysis have been investigated. Various computational tools like Molecular Evolutionary Genetics Analysis (*MEGA*) X and Figtree, Iterative Threading Assembly Refinement (I-TASSER), self-optimized predicted method with alignment (SOPMA), PROCHECK, University of California, San Francisco (UCSF) Chimera, and Search Tool for the Retrieval of Interacting Genes/Proteins (STRING) were used for characterization of cocoonase and sericin proteins. Sodium dodecyl sulfate-polyacrylamide gel electrophoresis (SDS-PAGE), protein purification using Sephadex G 25-column, degumming of cocoon sheet using cocoonase enzyme and chemical Na_2_CO_3_, and SEM and OCT analysis of degummed cocoon sheet were performed.

**Results:**

Predicted normalized B-factors of cocoonase and sericin with respect to α and β regions showed that these regions are structurally more stable in cocoonase while less stable in sericin. Conserved domain analysis revealed that *B. mori* cocoonase contains a trypsin-like serine protease with active site range 45 to 180 query sequences while substrate binding site from 175 to 200 query sequences. SDS-PAGE analysis of cocoonase indicated its molecular weight of 25–26 kDa. Na_2_CO_3_ treatment showed more degumming effect (i.e., cocoon sheet weight loss) as compared to degumming with cocoonase. However, cocoonase-treated silk cocoon sheet holds the natural color of tasar silk, smoothness, and luster compared with the cocoon sheet treated with Na_2_CO_3_. SEM-based analysis showed the noticeable variation on the surface of silk fiber treated with cocoonase and Na_2_CO_3_. OCT analysis also exemplified the variations in the cross-sectional view of the cocoonase and Na_2_CO_3_-treated silk sheets.

**Conclusions:**

Present study enlightens on the detailed characteristics of cocoonase and sericin proteins, comparative degumming activity, and image analysis of cocoonase enzyme and Na_2_CO_3_ chemical-treated silk sheets. Obtained findings illustrated about use of cocoonase enzyme in the degumming of silk cocoon at larger scale that will be a boon to the silk industry.

**Supplementary Information:**

The online version contains supplementary material available at 10.1186/s43141-021-00125-2.

## Background

Sericulture is an industry having a history of almost 5000 years where raising of silkworm larvae and turning of cocoons into a string is being performed. Domestic silk moth (*Bombyx mori*) is a holometabolous insect having four discrete stages (such as egg, larva, pupa, and adult) in their life cycle simply separated from one another [22]. The prevalence of silk as a material fiber has been perceived from the prehistoric time. Silkworm is generally nurtured in different geological locales of India. Arjuna or arjun tree (*Terminalia arjuna*) and asan or Indian laurel or silver-grey wood (*Terminalia tomentosa*) are primary host plants for the *A. mylitta* larvae. Jamun (*Syzygium cumini *L.) is another potential host of tropical Tasar silkworm, *A. mylitta* Drury. Tasar cocoons are tougher than coverings of other types of sericigenous insects. The silk fiber delivered by the silkworm is a complex material shaped by fibroin protein and bounded by sericin protein [45]. To get the silk thread from cocoons, removal of sericin is an essential step [52]. As an essential to reeling practice, cooking of cocoon needs to be performed. Here, cocoon is made softer by decomposing or partially solubilizing the sericin component that ties the protein fibroin strands from which the silk string is reeled.

*A. mylitta* insect species is native to India. This is an economical sericigenous insect that produces tasar silk having high demand worldwide. They are broadly distributed in the tropical area of India lies from West Bengal (East) to Karnataka (South). It is also found in the forest of Madhya Pradesh, Bihar, Maharashtra, Andhra Pradesh, Telangana, and Orissa [59]. Tasar silk obtained from *A. mylitta* species of wild silkworms is a different color from domesticated silkworm silk. It is coarser and stronger that makes it more favorable in some applications like higher tensile strength, elongation, and stress-relaxation values than the silk secreted by the domesticated silkworm *B. mori* [12, 28, 56]. The natural silks are extensively categorized as mulberry (which is obtained from cocoons of *B. mori* L.) and non-mulberry (which is obtained from tropical and eri, muga, temperate tasar, and anaphe). About 95% of the worldwide generation of non-mulberry silk belongs to tasar. Other varieties (like fagara, coan, mussel, and creepy crawly silks) are not utilized for profitable production [45]. Tasar silk fiber has its own unique shading, elongation, coarse to feel, higher elasticity and stress-relaxation values as compared to mulberry silk fiber. These properties have made tasar silk as capable and attractive as mulberry silk. *B. mori* has four discrete stages in their life cycle where only larva stage (i.e., from 1st to 5th larval instars) is a feeding period. Also, morphologically remarkable changes from larva to adult occur in the pupa by a wonderfully regulated metabolism that consist of the degradation, remodeling, and neogenesis of the tissues [22]. Reeling is an important method where silkworm is used for drawing silk thread from cocoon spun [45]. There is regularly expanding interest for tasar silk because of its luster, strength, and copper brown color.

In India, the production of tasar silk continued next to mulberry silk for eras, constituting about 4% of the total production of silk. The cocoon cooking involves boiling of the cocoon in water that helps in the release of sericin protein and a continuous silk filament that is reeled to get a thick thread of silk [9]. After degumming of fibers, the mulberry silk is soft, white, and holds luster while non-mulberry silk is irregular, coarser, and brownish in color. In industries, chemical methods are used for degumming of cocoons using chemicals like soda, soap, H_2_O_2_, alkaline solution, and alkali. Sericin and fibroin both are affected by chemical treatment, thus affect the properties of tasar silk-like natural color and softness [51]. Therefore, it is anticipated that enzyme-based cocoon degumming will be beneficial in sustaining the natural color and softness of tasar silk. Also, enzymatic methods have many advantages over chemical method as it is cheap, eco-friendly, and enriches the silk quality [17].

A couple of decades ago, it was realized that enzyme-based degumming of silk cocoon needs to be established because enzyme-based cocoon degumming results in a silk yarn having good texture and upgraded gloss. For enzyme-based degumming process, mainly papain, trypsin, and bacterial enzymes were used [31]. A proteolytic enzyme trypsin that is secreted by the pancreas catalyzes the hydrolysis of the peptide bond among the carboxyl group of lysine or the carboxyl group of arginine and amino groups of adjacent amino acids. Trypsin is mostly active at the temperature of 37 °C and in the pH range of 7–8. Sericin is a less crystalline protein with a comparatively high lysine and arginine content, polar in nature as well as effectively hydrolyzed by trypsin, while fibroin is not affected by trypsin because of a lower quantity of the arginine and lysine present in its structure [25]. An enzymatic method of degumming includes the use of proteolytic enzymes (like papain, bromelain, trypsin, alcalase, protease) that hydrolyzes the peptide bond of protein and degrades sericin without disturbing fibroin [18].

Cocoonases [enzyme commission (EC) number: 3.4.21.4] are sericin proteinases secreted by few sericigenous insects that soften the end part of the silk cocoon and allow to escape the adult moth [34]. Cocoonase is a proteolytic enzyme produced by silk moth during the pupal-adult transformation. Its main function is to digest the sericin protein at the anterior portion of the cocoon. Cocoonase enzyme is synthesized and collected in the maxillary galeae of silk insect as prococoonase [8, 19, 36, 38]. The SDS-PAGE based study of freshly collected cocoonase exhibited its molecular weight of 25–26 kDa [52].

Computational biology based phylogenetic analysis of cocoonase using the Molecular Evolutionary Genetics Analysis (MEGA) 5.1 Beta4 software showed the existence of conserved domain in cocoonase [43]. Current and future perspective of cocoonase enzyme indicating its detailed possible role in tasar industry have been elucidated [51]. Development of modern biotechnological and molecular biology tools has eased to know the detailed information about the gene and genome of any organism. However, much information of genes as well as whole genome sequence of the tasar silkworm *A. mylitta* is not yet available. A computational approach-based study by utilizing the all available expressed sequence tags (*ESTs*) towards predicting the microRNA (miRNA) and single nucleotide polymorphisms (SNPs) in *A. mylitta* has been reported [16]*.*

Detailed information on cocoonase and sericin proteins about their sequence characteristics and evolutionary relationship, structures, validation, and their interactions in *A. mylitta* are not available. Therefore, a holistic study on cocoonase and sericin using computational and experimental approaches is of great interest. In the present study, computational analysis has been executed using online *B. mori* sequences. Also, details about cocoonase collection, purification, degumming of silk cocoon shell and its effect on cocoon sheet showing microscopic differences in enzyme- and chemical-treated cocoon shell, and its characteristics using a scanning electron microscope (SEM) and optical coherence tomography (OCT) have been elucidated.

## Methods

### Sequence retrieval

Sequence retrieval of cocoonase (BAJ46146.1) and sericin (BAD00699.1) proteins in FASTA format was performed using the National Center for Biotechnology Information (NCBI) (https://www.ncbi.nlm.nih.gov/).

### BLAST

BLAST (basic local alignment search tool) is a sequence similarity search program. The retrieved cocoonase (GenBank ID: BAJ46146.1) and sericin (GenBank ID: BAD00699.1) proteins from NCBI were further searched for similarity checking in NCBI using BLASTP. BLASTP was performed against the query sequence to establish search for the template sequence with highest similarity. Algorithm was set at 250, and only cocoonase and sericin protein sequences of different species were selected.

### Phylogenetic tree

The phylogenetic tree was constructed to establish evolutionary relationship between the template and the sequence retrieved after BLAST analysis. Molecular Evolutionary Genetics Analysis (MEGA) X, an integrated tool for conducting automatic and manual sequence alignment, inferring phylogenetic trees and mining web-based databases (https://www.megasoftware.net/) was used to construct the tree [39]. Figtree that is designed as a graphical viewer of phylogenetic trees and as a program for producing publication-ready figures (http://tree.bio.ed.ac.uk/software/figtree/) was used to color the different species in phylogenetic tree.

### SOPMA

SOPMA (self-optimized predicted method with alignment) is mostly being used to analyze secondary structure of protein. Sequence length and secondary structure of cocoonase (BAJ46146.1) and sericin (BAD00699.1) proteins were predicted by SOPMA available at http://npsa-pbil.ibcp.fr/cgi-bin/npsa_automat.pl?page=/NPSA/npsa_sopma.html [23]. The improvement happens in the way that SOPMA considers data from an arrangement of successions having a place with a similar family.

### Structure prediction by I-TASSER

I-TASSER (Iterative Threading Assembly Refinement) is an online hierarchical approach and is used for the prediction of structure and function of the protein available at https://zhanglab.ccmb.med.umich.edu/I-TASSER/ [61, 74, 78]. Structure of cocoonase (BAJ46146.1) and sericin (BAD00699.1) proteins was predicted using online I-TASSER tool.

### Prediction of ligand-binding sites

Initially, I-TASSER model was submitted to the COACH algorithm available at https://zhanglab.ccmb.med.umich.edu/COACH/, which produces ligand-binding site predictions by matching the target models with the proteins in the BioLiP database [71, 72].

### Prediction of Enzyme Commission (EC) numbers and active sites

Predictions of Enzyme Commission number and active site were generated by COFACTOR and local and global structural evaluations of the I-TASSER models with known proteins in the BioLiP structure function database available at https://zhanglab.ccmb.med.umich.edu/COFACTOR/help.html [75].

### Prediction of normalized B-factor

B-factor (also known as temperature factor) is regularly used to know the extent of atomic motion in the X-ray crystallography. Here, the normalized B-factor was predicted by ResQ [73] using a combination of template-based assignment and machine-learning-based prediction which employs sequence profile and predicted structural features.

### Ramachandran plot by PROCHECK

Ramachandran plot is used for the validation of tertiary structure. Ramachandran plot was prepared for cocoonase and (https://www.ebi.ac.uk/thornton-srv/software/PROCHECK/) was used for validation of tertiary structure and “stereochemical quality” of a given protein structure.

### UCSF Chimera

Chimera is segmented into a core that offers elementary services and visualization, and extensions that provide most higher-level functionality [54]. Chimera is freely accessible to academic and nonprofit researchers and available at https://www.cgl.ucsf.edu/chimera/. University of California, San Francisco (UCSF) Chimera showed the good molecular visualization of the 3D models and was used to generate good quality images of the protein models. This software was used for the better visualization of the 3D structures of cocoonase and sericin proteins [49].

### Conserved Domain

Conserved Domain records the location of functional motifs on protein domain models, so that these motifs can be mapped on protein sequences and facilitate the interpretation of sequence conservation and variation in active sites, chemical binding, and protein-protein interaction sites. Cocoonase (BAJ46146.1) and sericin (BAD00699.1) proteins were used to predict the presence of conserve domain available at https://www.ncbi.nlm.nih.gov/Structure/cdd/cdd.shtml [47, 48].

### Networking of proteins

A functional interacting network of cocoonase (BAJ46146.1) and sericin (NP_001037329.1, SGF1 -Silk gland factor 1; regulates the transcription of the sericin-1 gene via interaction with the SA site) proteins was performed for the protein sequences using the Search Tool for the Retrieval of Interacting Genes/Proteins (STRING) 10 software [21, 66].

### Recognition of specific stage of pupae

Specific stage of pupae that may be utilized for maximum cocoonase collection was recognized. These pupae were kept for adult emergence and monitored by changes in integument color from natural red-brownish to black [53].

### Collection of proteolytic enzyme cocoonase

Freshly pierced cocoons were taken, and before emergence, pupae were transferred to cocoonase collection set-up for cocoonase secretion and collection [53]. Briefly, pupae were kept in a funnel that facilitates collection of secreted cocoonase in a small test tube embedded in ice to keep the collected cocoonase at lower temperature.

### Degumming activity assessment of cocoonase

Degumming activity assessment of cocoonase was performed as per method described previously by Wang and Guo [68] with modifications. For this analysis, silk cocoon sheets (each 30 mg in weight) were dried using hot air oven to remove the moisture completely. Degumming activity was studied in three different test tubes, namely, (a) control-silk cocoon sheet dissolved in 10 ml of Tris-HCl (pH 8.0) buffer; (b) cocoonase enzyme degumming-silk cocoon sheet dissolved in 0.2 ml of cocoonase enzyme + 10 ml of Tris-HCl (pH 8.0) buffer; and (c) Na_2_CO_3_-based alkaline degumming–silk cocoon sheet dissolved in 0.05% of Na_2_CO_3_. All three test tubes were incubated at 42 °C for 1 h with agitation. Silk cocoon sheets were rinsed with warm water followed by distilled water thrice. All three treated cocoon sheets were dried for 1 day at ambient temperature followed by at 70 °C for 1 h. Subsequently, dry weight was measured using analytical balance. Experiment was performed using three independent replications, and obtained values were averaged.

### Cocoonase quantification and purification

The estimation of protein was performed according to Bradford’s protein assay method [10]. The protein sample concentration was determined from a standard curve drawn using bovine serum albumin as a standard. Crude cocoonase was purified using Sephadex G 25-column.

### SDS-PAGE analysis

The sodium dodecyl sulfate-polyacrylamide gel electrophoresis (SDS-PAGE) analysis was carried out according to the procedure of Laemmli [40] with slight modification. Six percent stacking gel with pH 6.8 while 10%, 12%, and 15% of resolving gel with pH 8.8 was used. Tris-glycine with 0.1% SDS having pH 8.6 was used as running buffer. A total of 10 μl of cocoonase was boiled for 10 min with equal volume of 1X protein loading buffer. After boiling, the protein samples were immediately chilled on ice for 10 min. These samples were loaded in gel, and the resolved protein was visualized by Coomassie blue staining as per standardized protocol.

### Scanning electron microscopy observation of silk filaments

Silk cocoon sheet degummed only in Tris-HCl buffer (control) and sheet obtained after degumming with cocoonase enzyme as well as sheet treated with Na_2_CO_3_ were used for morphological observation by using a scanning electron microscope (SEM). Comparative morphological analysis of silk sheets subjected to varying treatment was performed.

### Optical coherence tomography-based analysis

Silk cocoon sheets treated only with buffer (control), cocoonase enzyme, and chemical were subjected to OCT system and imaged [58]. For this analysis, all three test tubes (control, treated with cocoonase enzyme, treated with chemical) having cocoon sheets were incubated at 42 °C for 1 h only. After treatment, silk sheets were rinsed with warm water followed by distilled water thrice. All three sheets were dried for 1 day at ambient temperature followed by at 70 °C for 1 h. Subsequently, these treated and dried sheets were used for image acquisition. Image acquisition was carried out the next day after the treatment. To minimize the moisture content, treated cocoon sheets were stored in an incubator at 37 °C. Finally, these sheets were mounted on microscope slides for recording observations and comparative analysis using the OCT-inbuilt software.

## Results

**Fig. 1:**
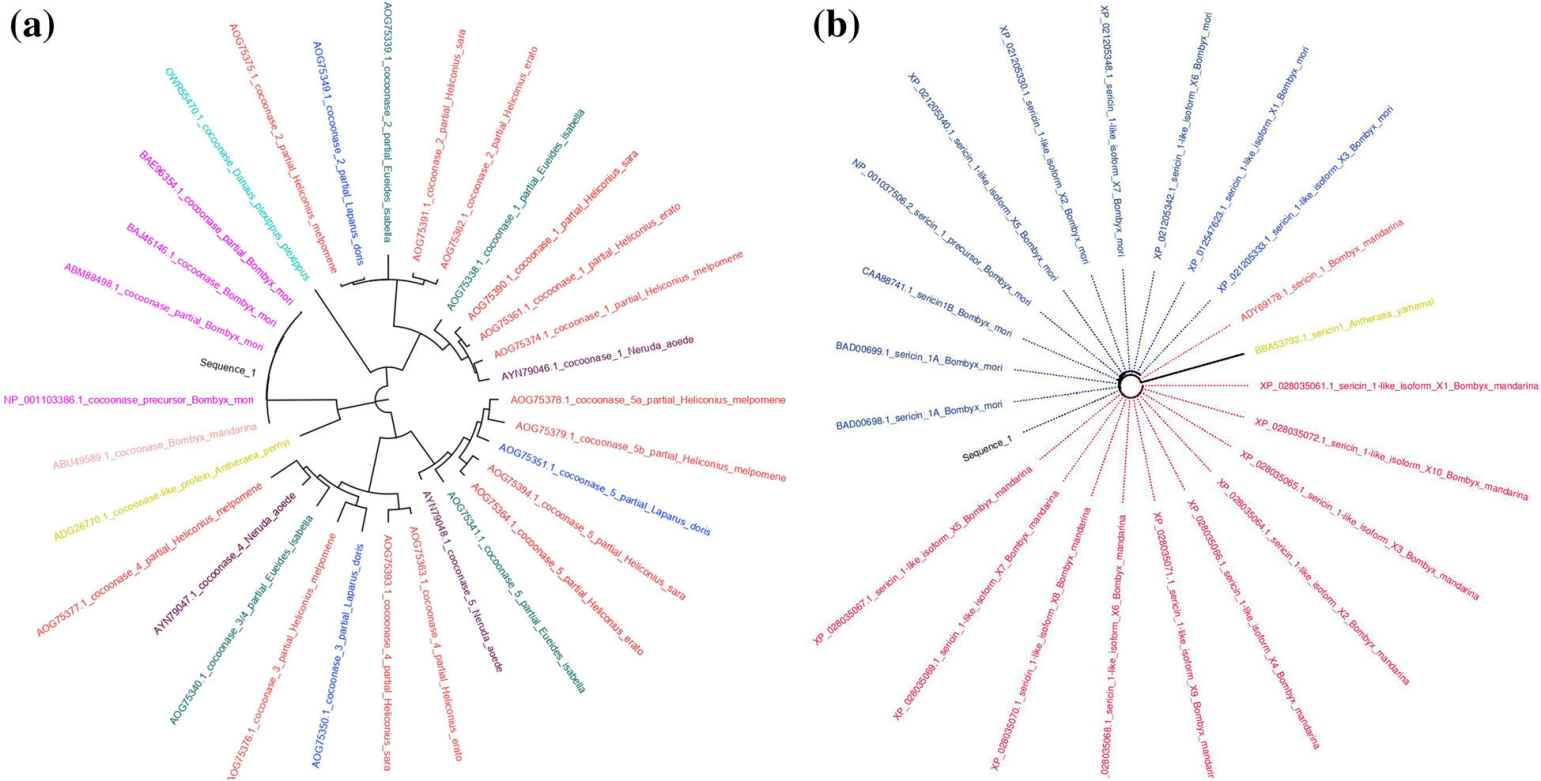
The phylogenetic tree of cocoonase (**a**) and sericin (**b**) that was constructed using MEGA X [39]. The evolutionary history was inferred by using the Maximum Likelihood method and JTT matrix-based model [32]. The tree with the highest log likelihood of − 5997.87 is shown in (**a**) and − 10557.91 in (**b**)

**Fig. 2:**
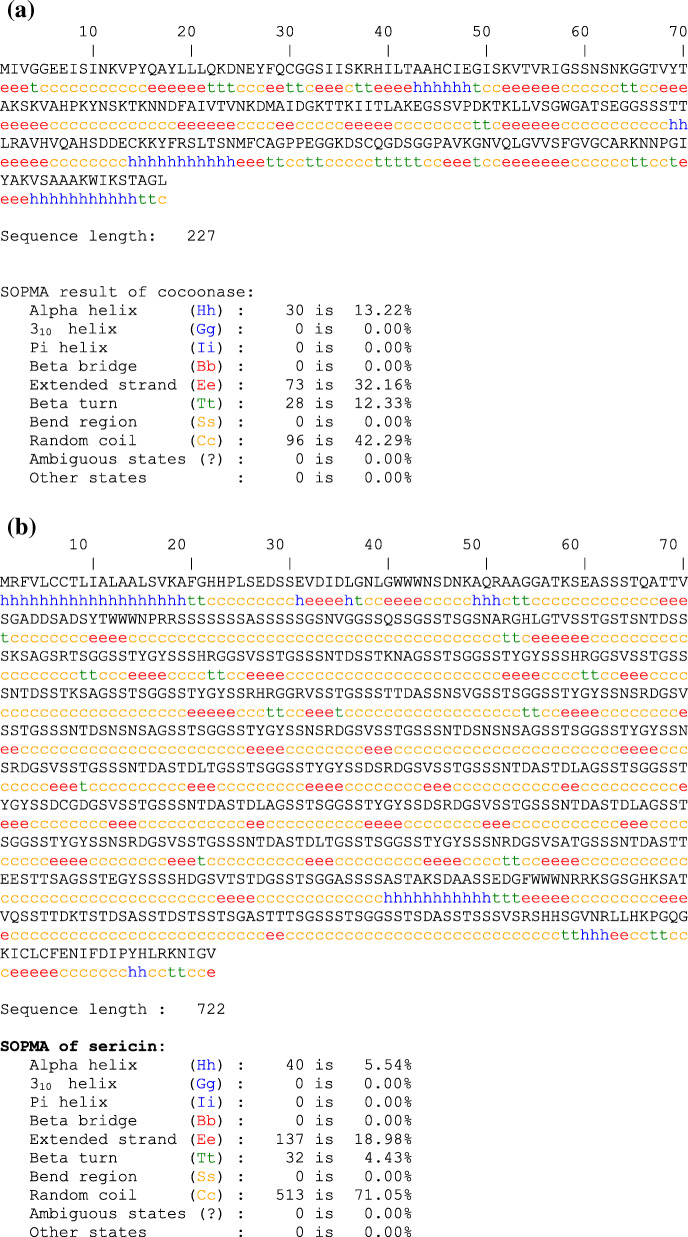
Secondary structure prediction of cocoonase (**a**) and sericin (**b**) using SOPMA server

Sequence of cocoonase (BAJ46146.1) and sericin 1A’ (BAD00699.1) proteins from *Bombyx mori* having amino acid query length of 227 and 722, respectively, was retrieved from NCBI. Phylogenetic analysis of all the retrieved protein sequences exhibited evolutionary relationship among different species. Evolutionary relationship was shown by using the Maximum Likelihood method and JTT matrix-based model. Different cocoonases of *B. mori* showed substantial similarity among each other as compared to cocoonase from other species. Various sericin proteins of *B. mori* also showed noteworthy similarity among each other as compared to sericin of other species. A total of 32 amino acid sequences of cocoonase and 23 amnio acid sequences of sericin protein were found and considered for analysis (Fig. 1 a and b). There were a total of 276 positions in the final dataset of cocoonase and 1953 positions in the final dataset of sericin (Fig. 2 a and b and Supplementary Figure 1a & b).

**Fig. 3:**
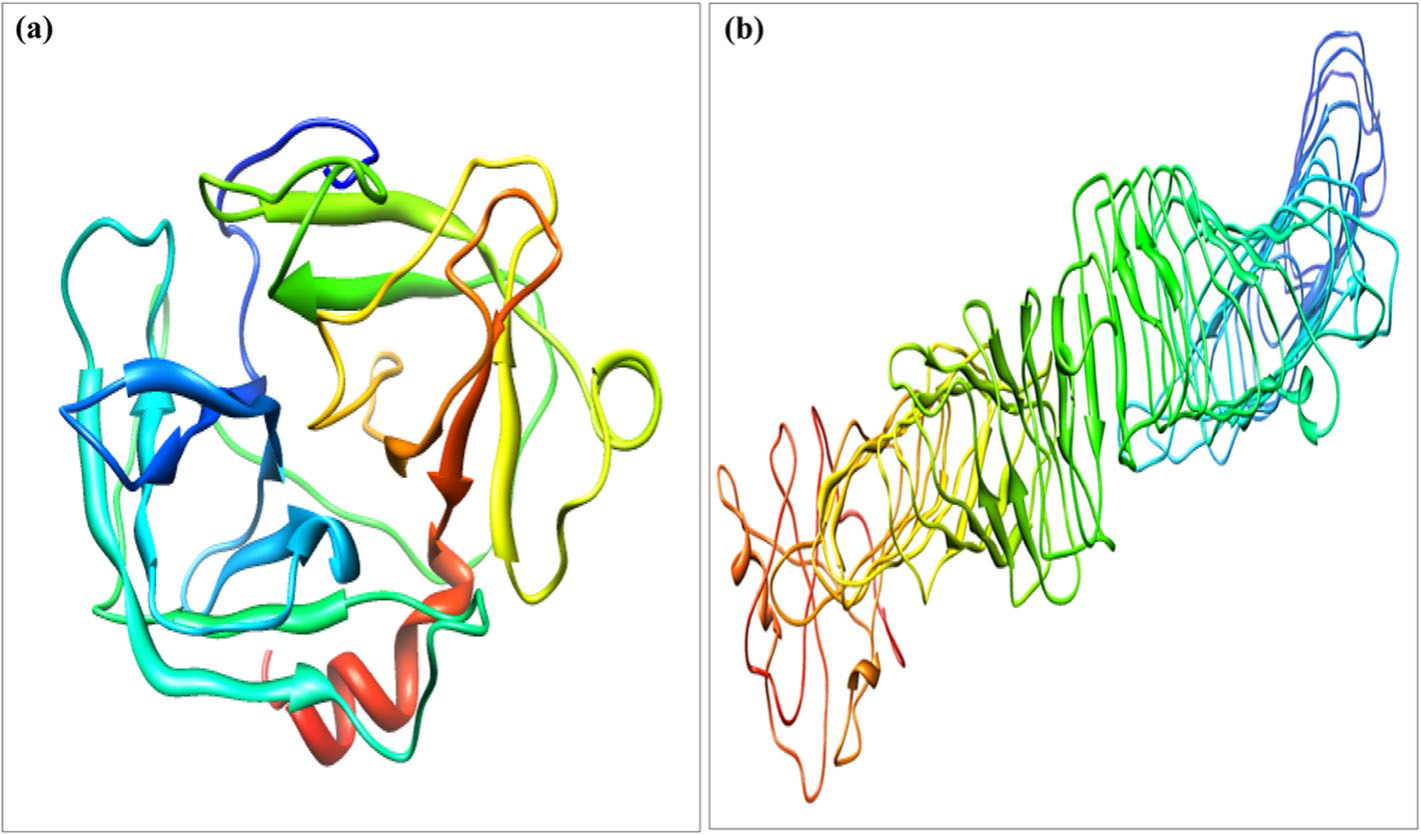
Predicted 3D structure of cocoonse and sericin. **a** Cocoonase with C-score 1.19 showing the model of correct global topology, and **b** sericin with C-score − 0.53 and C-score > − 1.5 indicating a model of correct global topology. The models are colored based on rainbow coloring scheme with N-terminal of protein colored blue and C-terminal colored red. For cocoonase and sericin, the helical structures in red color represent α-helix, 3_10_-helical structures in blue color, and arrow in yellow color represent β-sheets; turns were represented in cyan, and coils were in purple color

**Fig. 4:**
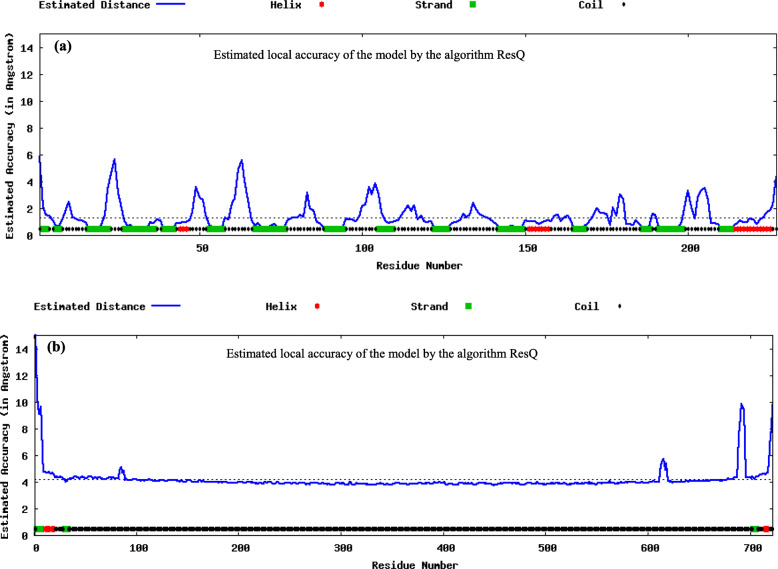
Cocoonase (**a**) and sericin (**b**) showing the local accuracy estimation for the first model predicted by I-TASSER. Also, majority of residues in the models are modeled accurately with estimated distance to native below 2 A° for cocoonase and below 6A° for sericin

I-TASSER produces a full-length model of proteins by removing continuous fragments from threading alignments and afterward reassembling them utilizing replica-exchanged Monte Carlo simulation. The models are colored based on rainbow coloring scheme with N-terminal of protein colored blue and C-terminal colored red. The helical structures in red color represent α-helix, 3_10_-helical structures in blue color, and arrow in yellow color represents β-sheets whereas turns were represented in cyan and coils in purple color (Fig. 3 a and b). After the simulation of structure assembly, I-TASSER utilizes the TM-align structural alignment program to match the major I-TASSER model to all the structures in the Protein Data Bank (PDB) library. This segment reports the main 10 proteins from the PDB showing the nearest basic closeness, i.e., the highest TM-score to the predicted I-TASSER model. Predicted tertiary structure of cocoonase exhibited the C-score 1.19, estimated TM-score 0.88 ± 0.07, and estimated RMSD 3.2 ± 2.3 Å, while sericin displayed the C-score − 0.53, estimated TM-score 0.65 ± 0.13, and estimated RMSD 3.2 ± 2.3 Å (Fig. 3 a and b). ResQ-based local accuracy estimation for the first model predicted by I-TASSER for cocoonase and sericin proteins was also analyzed. Result showed that majority of residues in the models were modeled accurately with estimated distance to native below 2 A° for cocoonase and below 6 A° for sericin (Fig. 4 a and b).

**Fig. 5:**
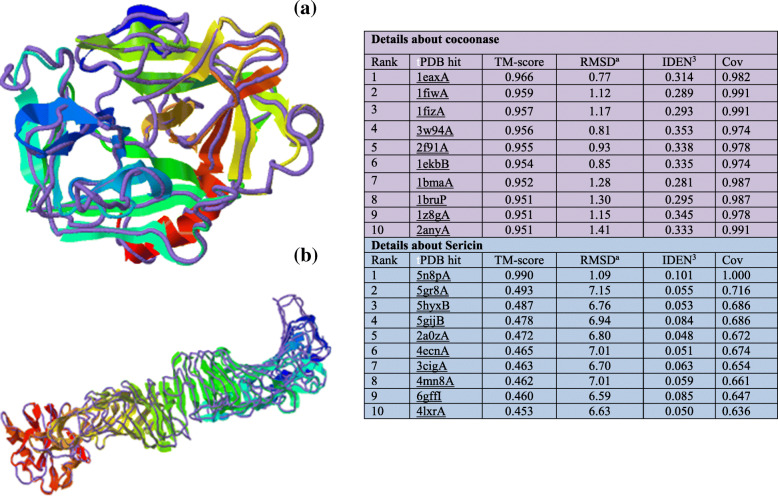
The structure alignment of both proteins like cocoonase (**a**) and sericin (**b**), between the first I-TASSER model and the top 10 most similar structure template in PDB. Ten PDB structures were predicted nearby to the target. The structure of the first I-TASSER model (shown in rainbow cartoon) is superimposed on the parallel structures from the PDB (shown in purple backbone touch). The structure similarity between the objective model and the 10 nearest proteins is positioned by TM-scores

Here, top 10 proteins from the PDB showing the closest structural similarity, i.e., the highest TM-score to the predicted I-TASSER model, were reported. Total 10 PDB hits (1eaxA, 1fiwA, 1fizA, 3w94A, 2f91A, 1ekbB, 1bmaA, 1bruP, 1z8gA, 2anyA) for cocoonase and 10 PDB hits (5n8pA, 5gr8A, 5hyxB, 5gijB, 2a0zA, 4ecnA, 3cigA, 4mn8A, 6gffI, 4lxrA) for sericin protein have been observed. PDB hit 1eaxA for cocoonase, while 5n8pA for sericin, were top ranked proteins based on TM-score of the structural alignment between the query structure and known structures in the PDB library. RMSD^a^ indicating the RMSD between structurally aligned residues by TM-align for these top-ranked proteins of cocoonase (1eaxA) and sericin (5n8pA) were minimum with the value of 0.77 and 1.09, respectively (Fig. 5 a and b).

**Fig. 6:**
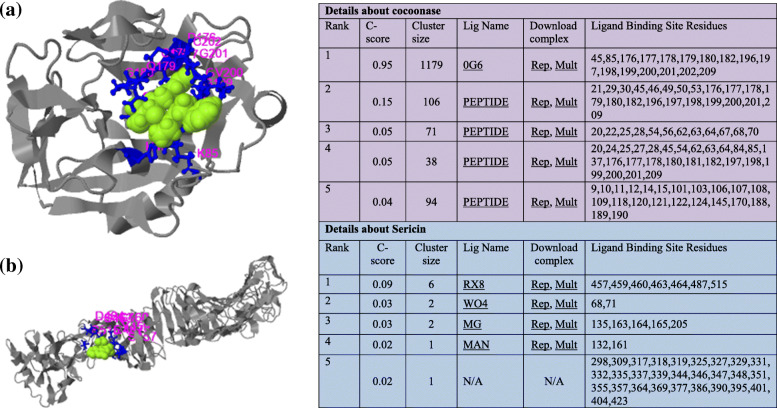
The predicted ligand-binding sites in cocoonase (**a**) and sericin (**b**). The first functional template (PDB ID: 5jbcS) has a high confidence score (C-score = 0.95) for cocoonase which is showing the structure is stable and functional. The template (PDB ID: 3w3lA) for sericin has C-score of 0.09 which is also stable structure and will bind with a peptide ligand. But for the predicted peptide, the protein can likewise tie to different ligands, which are available in a PDB file at the “Mult” link

**Fig. 7:**
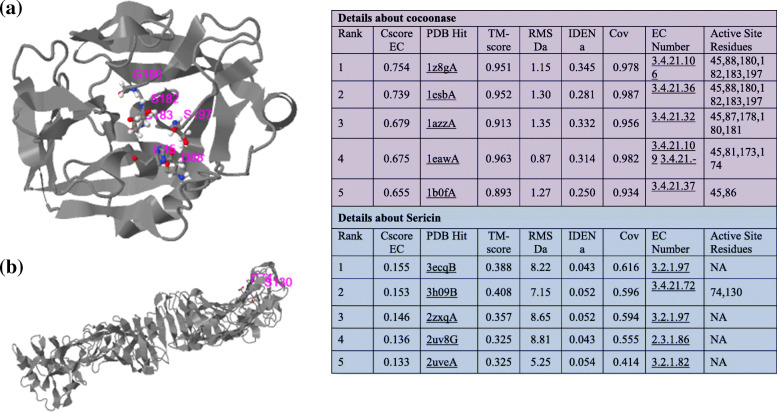
Predicted Enzyme Commission numbers and active sites for cocoonase (**a**) and sericin (**b**). The first model is predicted based on the template of PDB ID: 1z8gA. The predicted active-site residues are 45, 88, 180, 182, 183, and 197 shown in colored ball-and-sticks for cocoonase having C-score of 0.75, which is showing solid EC number. While for PDB ID: 3h09B, the predicted active-site residues are 74 and 130 shown in colored ball-and-sticks for sericin having C-score of 0.153, which is also predicting solid EC number

**Fig. 8:**
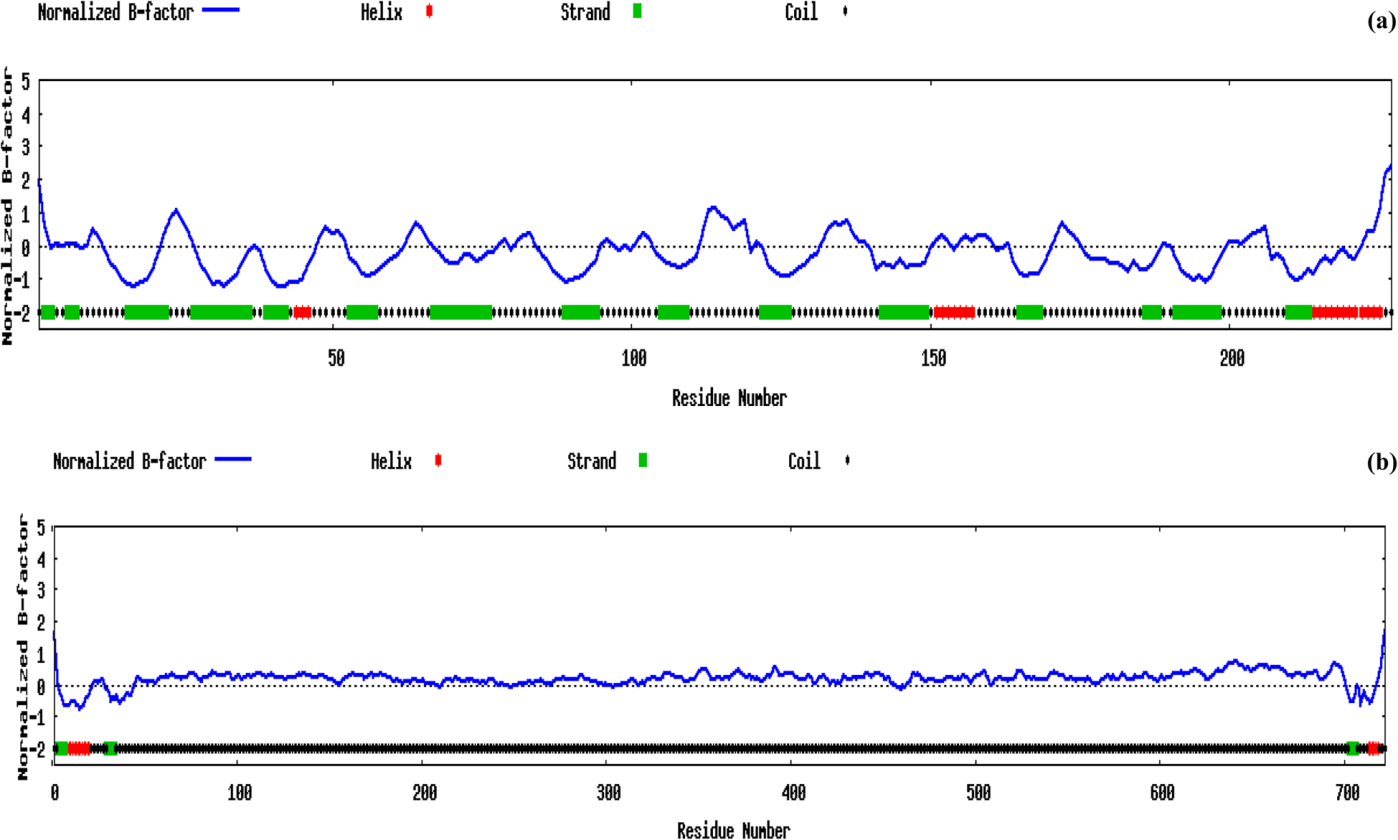
The predicted normalized B-factor by ResQ, showing the estimated global and local accuracy. The regions at the N- and C-terminals and most of the loop regions are predicted with positive normalized B-factors, indicating that these regions are structurally more flexible than other regions. **a** The predicted normalized B-factors of cocoonase for the α and β regions are negative or close to zero, suggesting that these regions are structurally more stable. **b** On the other hand, the predicted normalized B-factors of sericin for the alpha and beta regions are close to zero, suggesting these regions are structurally less stable

Biological annotations of the target proteins based on the I-TASSER structure prediction has been studied where COFACTOR deduces protein functions (like ligand-binding sites and EC number) using structure comparison and protein-protein networks. The functional templates of cocoonase (PDB ID: 5jbcS) as well as sericin (PDB ID: 3w3lA) with a high confidence score, C-score (0.95 and 0.09, respectively), have been predicted along with varying cluster size (i.e., total number of templates in a cluster), ligand name as well as ligand-binding site residues (Fig. 6 a and b). COFACTOR-based protein function prediction using structure, sequence, and protein-protein interaction properties has also been studied for finding Enzyme Commission number (EC) and ligand-binding sites [77]. COFACTOR tool-based analysis of cocoonase protein predicted a template of PDB ID:1z8gA having EC number 3.4.21.106 (a hepsin belonging to peptidase family S1A). Also, the predicted active-site residues were 45, 88, 180, 182, 183, and 197 (shown in colored ball-and-sticks) with a C-score of 0.75 indicating a solid EC number (Fig. 7a). Similarly, for sericin protein, another template PDB ID: 3h09B having EC number 3.4.21.72 (a IgA-specific serine endopeptidase belonging to peptidase family S1A) was predicted. Also, predicted active-site residues were 74 and 130 (shown in colored ball-and-sticks) with a C-score 0.153 also indicating a solid EC number (Fig. 7b). The normalized B-factor (also known as B-factor profile, BFP) was predicted by using a combinatorial approach of both template-based assignment and profile-based prediction where residues with BFP values higher than 0 were less stable in experimental structures [73]. In the present study, predicted normalized B-factors of cocoonase for the helix and strand regions were negative or close to zero (Fig. 8a). On the other hand predicted normalized B-factors of sericin for the helix and strand regions were close to zero (Fig. 8b).

**Fig. 9:**
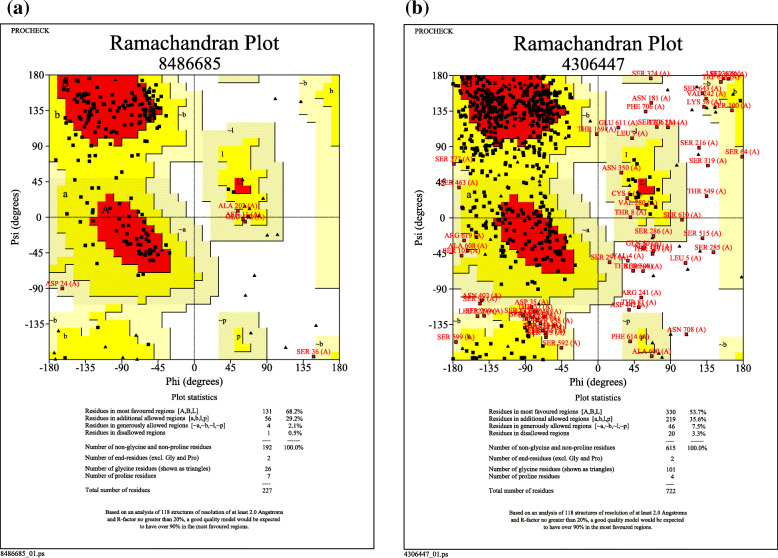
A Ramachandran plot output (modified from PROCHECK) of cocoonase (**a**) and sericin (**b**). The plot calculations were computed by PROCHECK server. The red regions in the graph indicate the most allowed regions; additional allowed, generous allowed, and disallowed regions are indicated as yellow, light yellow, and white fields, receptively. The Ramachandran plot for cocoonase disclosed 68.2% of amino acid residues within the most favored region. Similarly, Ramachandran plot for sericin disclosed 53.7% of amino acid residues within the most favored region

**Fig. 10:**
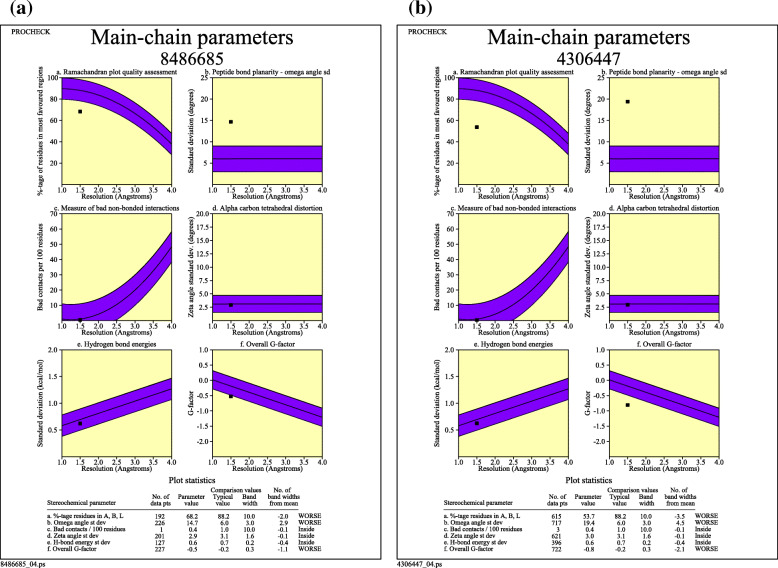
The plot cocoonase (**a**) and sericin (**b**) showing graphs of five main-chain properties of the structure and how these properties compared with well-refined structures at a similar resolution. In each graph, the dark band represents the results from the well-refined structures; the central line is a least-squares fit to the mean trend as a function of resolution, while the width of the band either side of it corresponds to a variation of one standard deviation about the mean

PROCHECK specified the stereochemical quality of a protein structure by analyzing residue-by-residue geometry and overall structure that analyzes the compatibility of an atomic model (3D) with its own amino acid sequence. A Ramachandran plot output (modified from PROCHECK) of cocoonase and sericin has been predicted (Fig. 9 a and b). The red region designated the most allowed regions, while yellow, light yellow, and white fields designate the additional allowed, generous allowed, and disallowed regions, respectively. The Ramachandran plot revealed that 68.2% amino acid residues of cocoonase and 53.7% amino acid residues of sericin were predicted within the most favored region (Fig. 9 a and b). To assess the geometric correctness of the theoretical structure, PROCHECK [42] was used to check the stereochemical quality of cocoonase and sericin residue-by-residue geometry. Plot of cocoonase (Fig. 10a) and sericin (Fig. 10b) indicated the graphs of five main-chain properties of their structures. In each graph, the dark band represented the results from the well-refined structures; the central line was a least-squares fit to the mean trend as a function of resolution, while the width of the band either side of it corresponds to a variation of one standard deviation about the mean [4]. In the present study, Ramachandran plot quality measured by the percentage of the protein’s residues that were in its most favored or core regions is indicated as (a), planarity of the peptide bond as measured by the standard deviation of the w torsion angles indicated as (b), number of bad contacts per 100 residues indicated as (c), tetrahedral distortion, measured by the standard deviation of the ~ zeta torsion angle indicated as (d), and the standard deviation of the hydrogen-bond energies for main-chain hydrogen bonds calculated using the method of Kabsch and Sander [33] has been indicated as (e).

**Fig. 11:**
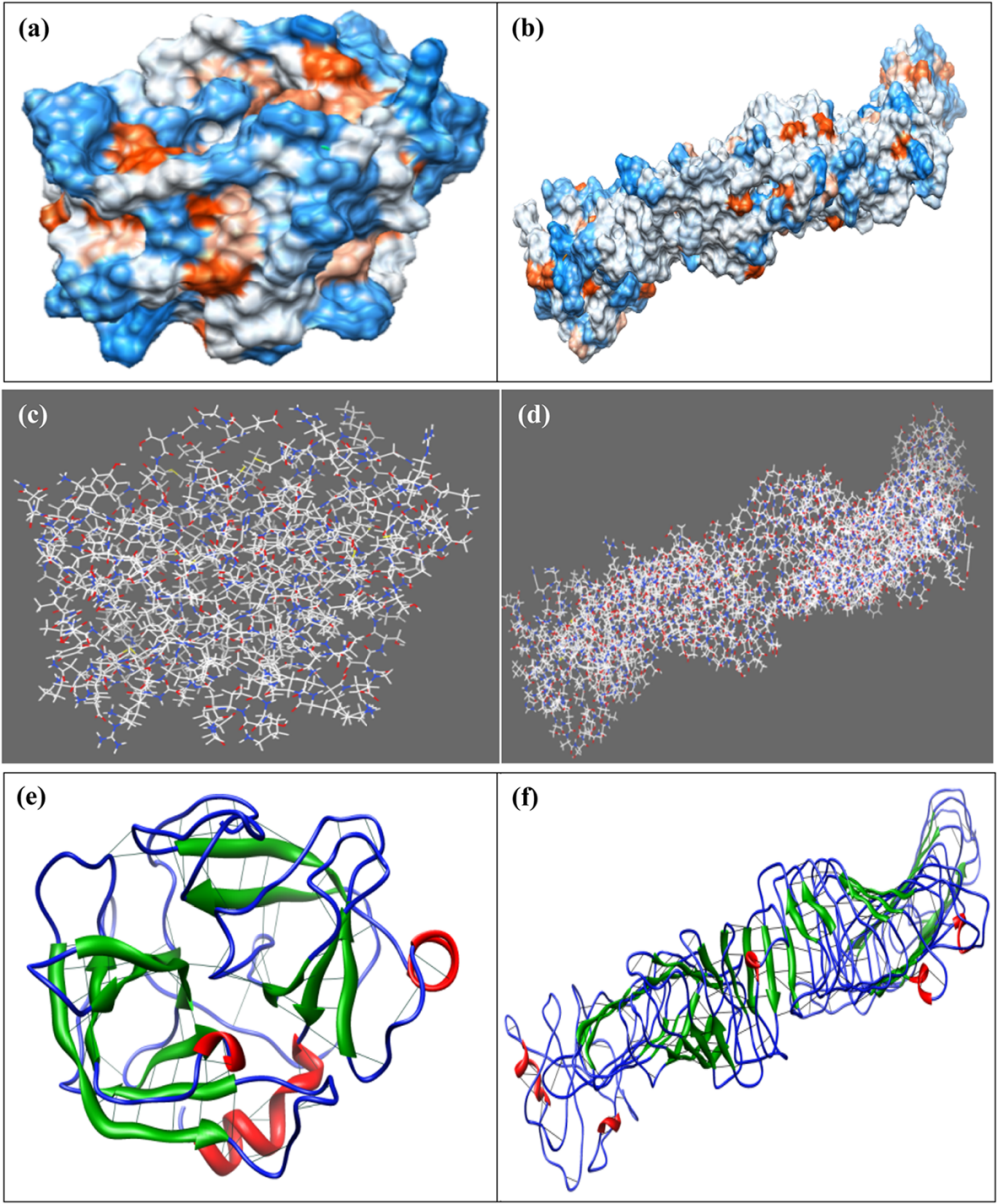
Cocoonase (**a**) and sericin (**b**) showing UCSF Chimera predicted the hydrophobicity of the protein. Blue color is showing the hydrophilic part of the protein, and orange color is showing the hydrophobic part of the protein. Cocoonase (**c**) and sericin (**d**) showing UCSF Chimera predicted every single atoms of the protein. Cocoonase (**e**) and sericin (**f**) showing UCSF Chimera predicted hydrogen bond in the protein models predicted by I-TASSER

Prediction of the hydrophobic and hydrophilic regions of the protein, single atom, and hydrogen bond in the protein models of cocoonase and sericin, which has been predicted by I-TASSER, were performed by UCSF Chimera. Here, blue color indicated the hydrophilic part of the protein while orange color indicated the hydrophobic part of the protein. Also, higher positive values correspond to more hydrophobic residues, and negative values correspond to hydrophilic residues. On the other hand, no-value color referring to residues lacking Kyte-Doolittle hydrophobicity (i.e., they are not amino acids such as the ligands in this structure) have been shown (Fig. 11 a and b). UCSF Chimera has been used to predict every single atom of the cocoonase and sericin proteins (Fig. 11 c and d) and hydrogen bond in the protein models of cocoonase and sericin (Fig. 11 e and f).

**Fig. 12:**
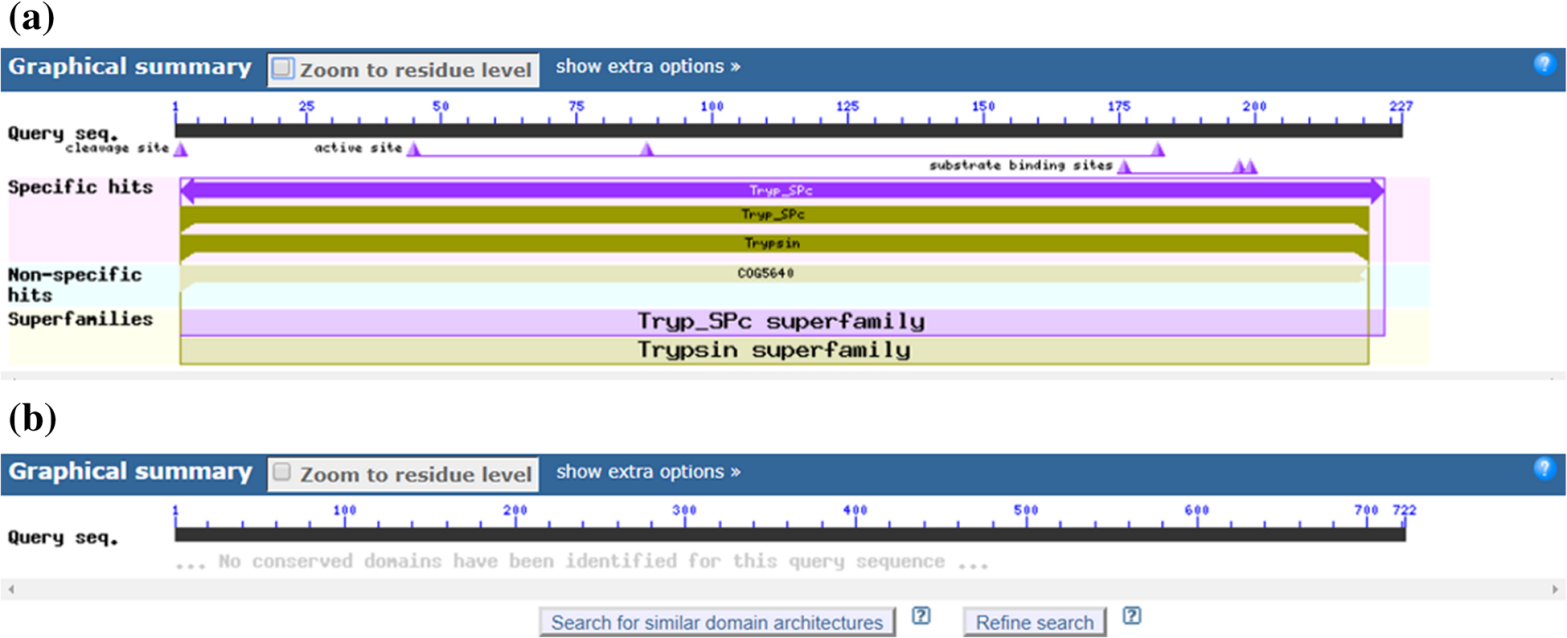
**a** Prediction of conserved domains of cocoonase [*Bombyx mori*] on [gi|315258604|dbj|BAJ46146.1|] (serine protease with domain architecture ID 10076129) trypsin-like serine protease. Many of these are synthesized as inactive precursor zymogens that are cleaved during limited proteolysis to generate their active forms. However, a few are active as single chain molecules while others are inactive due to substitutions of the catalytic triad residues [47, 48]. Here, pfam00089 [specific hit, evalue = 2.53e−59, trypsin] and COG5640 [non-specific hit, evalue = 9.80e−25, secreted trypsin-like serine protease] were predicted. **b** Prediction of conserved domains of sericin [*Bombyx mori*]. No conserved domain was predicted

**Fig. 13:**
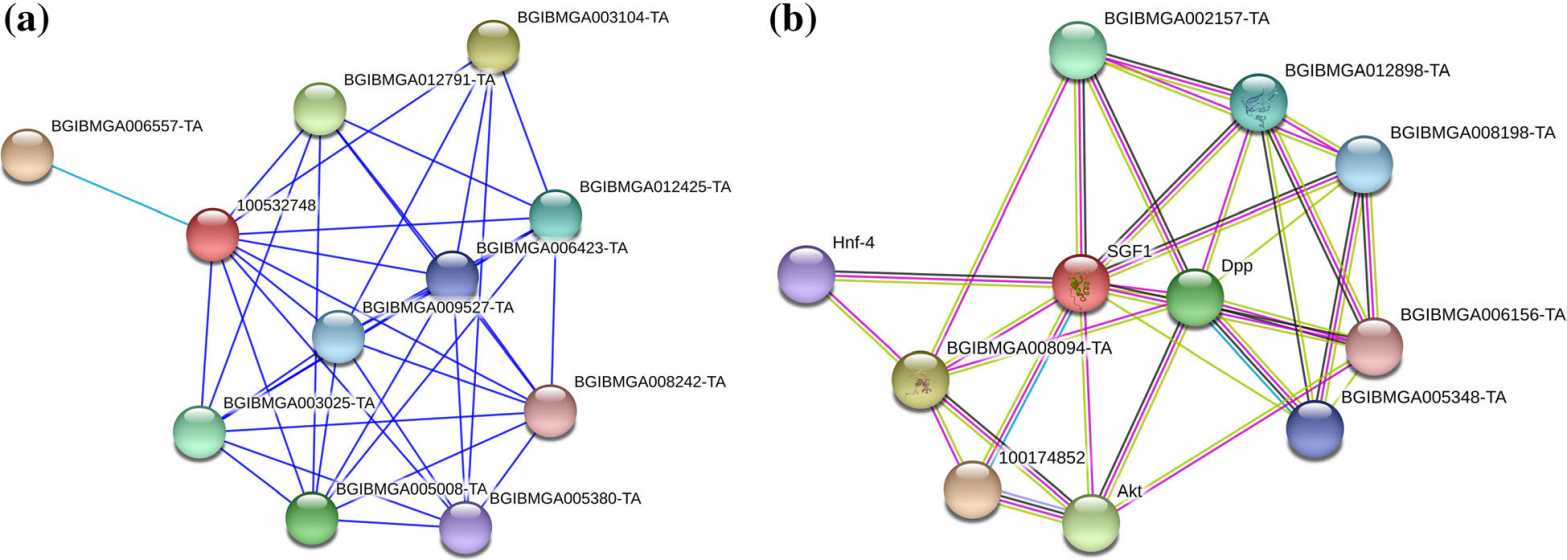
STRING analysis for cocoonase (BAJ46146.1, **a**) and sericin (NP_001037329.1, SGF1-Silk gland factor 1; regulates the transcription of the sericin-1 gene via interaction with the SA site, **b**) proteins. Line color indicates the type of interactions; line thickness indicates the strength of data support; line shape indicates the predicted mode of action; network nodes represent proteins. Each node represents all proteins produced by a single protein encoding gene locus. Colored nodes represent query proteins of first shell of interactors. White nodes represent second shell of interactors. Empty nodes represent proteins of unknown 3D structure. Filled nodes represent some 3D structure is known or predicted. Edges represent protein–protein associations.

Domains are related with protein structure, and therefore, prediction of domain might be useful in inferring protein function. Considering their importance, conserved domains were predicted in cocoonase and sericin proteins. Result indicated that conserved domains of *B. mori* cocoonase (Fig. 12a) were a trypsin-like serine protease having active site from 45 to 180 query sequence and substrate binding site from 175 to 200 query sequence while conserved domain of sericin (Fig. 12b) indicated no conserved domain availability in NCBI. To know about various other proteins that might be interacting with cocoonase and sericin proteins towards performing the specific functions and predicting their association in other biological events via protein-protein interacting network, a STRING database-based analysis was performed. A functional interacting network of cocoonase (BAJ46146.1) protein has been obtained (Fig. 13a). However, no functional interacting network of sericin (BAD00699.1) protein with other protein was predicted by STRING v10. Therefore, another sericin protein (NP_001037329.1, SGF1-Silk gland factor 1; regulates the transcription of the sericin-1 gene via interaction with the SA site from *Bombyx mori*) was used for STRING v10 analysis indicating the interaction with other proteins.

**Fig. 14:**
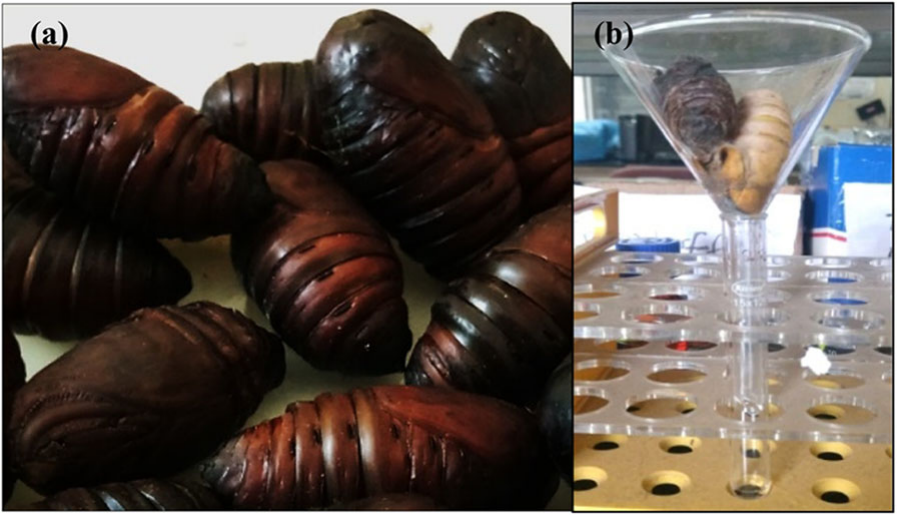
Identified suitable stage of pupae for cocoonase collection based on change in integument color (**a**) and collection of cocoonase enzyme setup (**b**)

**Fig. 15:**
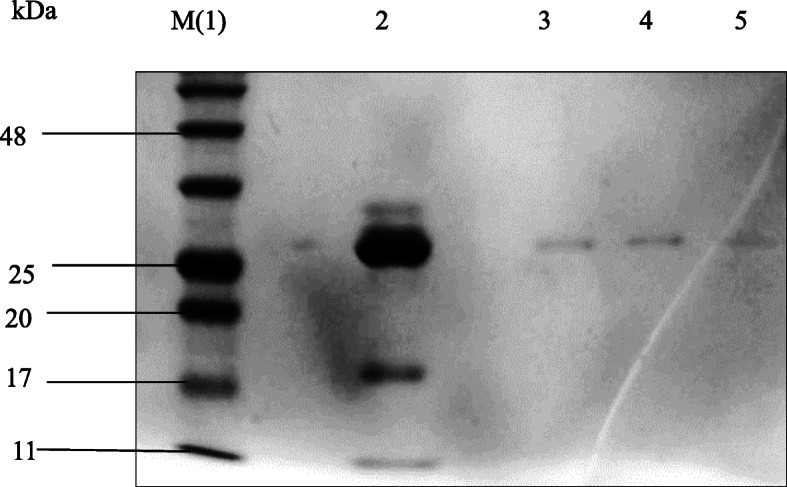
Purification of *A. mylitta* native cocoonase and its SDS-PAGE separation. Twelve percentage SDS-PAGE analysis showing 25–26 kDa cocoonase purified using Sephadex G25 column. Lane 1 (M) showing prestained protein marker of Himedia; lane 2 showing collected crude cocoonase, and lanes 3, 4, and 5 showing the purified cocoonase obtained in different fractions

To isolate and collect the maximum cocoonase, it was very much important to find the most suitable stage at which maximum enzyme is being released. Selection of pupae for cocoonase collection was typically based on change in the color of integument that turns dark black at the time of metamorphosis as well as softening of pupae tissues (Fig. 14a). Cocoonase is a proteolytic enzyme that is secreted by several sericigenous insect including *A. mylitta* during emergence. An emerging adult exudes around 500–850 μl of cocoonase gradually drop-by-drop, and this release process proceeds up to 2–4 h (Fig. 14b). Our result (Table 1) showed the cocoonase activity assessment in terms of comparative analysis of silk cocoon sheet weight treated with buffer (control), subjected to cocoonase treatment (enzyme degumming), and treated with Na_2_CO_3_ (chemical). The result showed degumming percentage in terms of decreased cocoon sheet weight treated with buffer, cocoonase enzyme, and Na_2_CO_3_ chemical. Result indicated that chemical-based degumming showed maximum degumming effect (cocoon sheet weight loss) as compared to degumming with cocoonase. SDS-PAGE-based protein separation of collected cocoonase contains many proteins with molecular weight of 29 kDa, 25–26 kDa, and 17 kDa proteins. However, Sephadex G25 column-based purification of cocoonase indicated its molecular weight of 25–26 kDa (Fig. 15).

**Table 1:** Degumming effect of cocoonase on cocoon sheet for 1 h at 42 °C

Treatment	Negative Control	Enzymatic degumming	Alkaline degumming
Sample	Cocoon sheet + buffer	Cocoon sheet + buffer + collected cocoonase	Cocoon sheet + Na_2_CO_3_ + buffer
Cocoon sheet weight added to each tube	30 mg	30 mg	30 mg
Cocoon sheet weight after complete drying at 70 °C (W_0_)	28.5 mg	28.8 mg	28.3 mg
Cocoon sheet weight after respective treatments at 42 °C (W_1_)	27.7 mg	26.9 mg	26.2 mg
$\mathrm{Degumming}\ \mathrm{ratio}\ \left(\%\right)=\frac{100\left({\mathrm{W}}_0-{\mathrm{W}}_1\right)}{{\mathrm{W}}_0}$	2.80	6.59	7.42

(1) Negative Control, i.e., cocoon sheet was treated with buffer only. Percentage loss of sericin in terms of decrease in cocoon sheet weight after the respective treatment was calculated. (2) Natural cocoonase degumming, i.e., cocoon sheet was treated with collected crude cocoonase enzyme. (3) Alkaline degumming, i.e., cocoon sheet was treated with Na_2_CO_3_

**Fig. 16:**
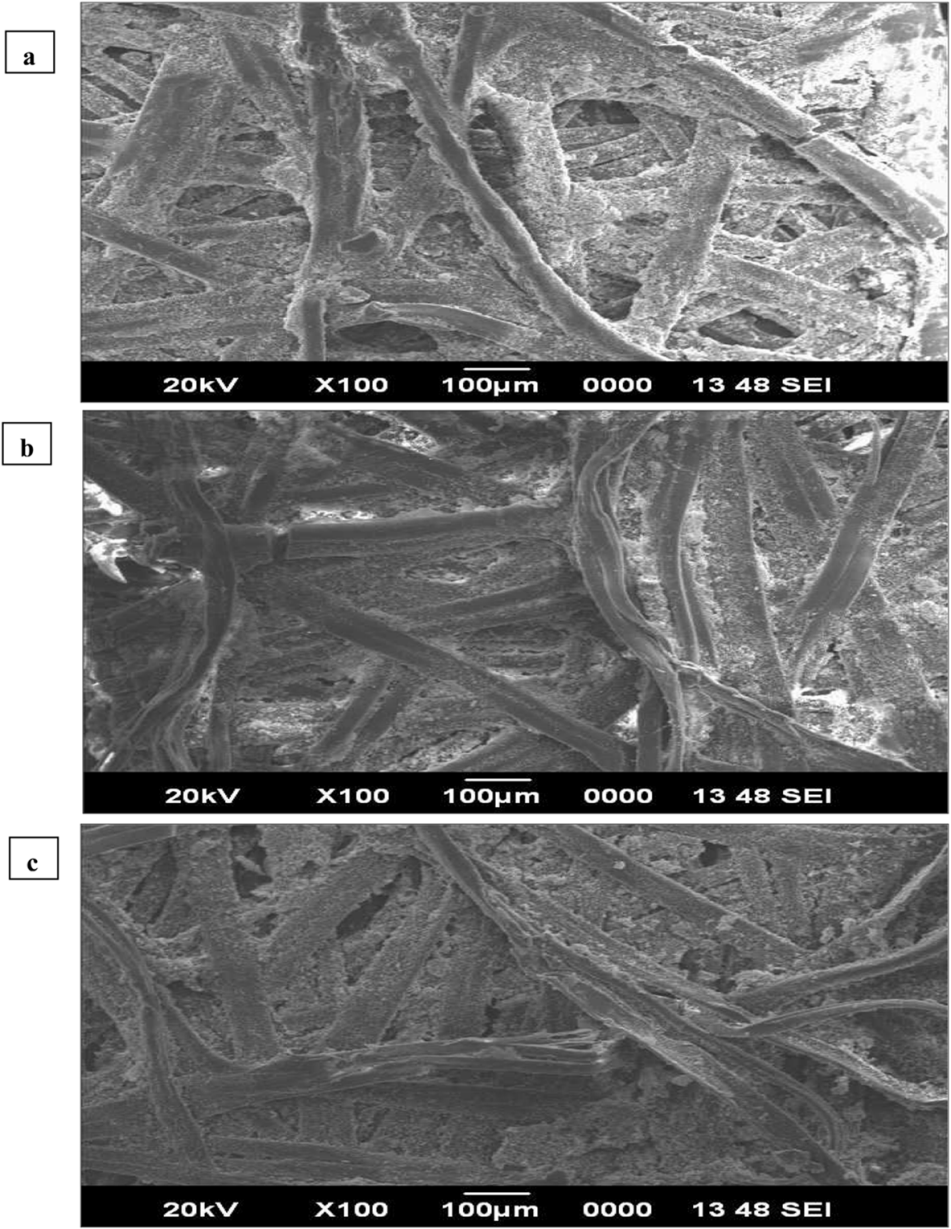
SEM image of silk sheet showing surface morphology. Surface morphology of silk sheet after cocoon treatment with buffer (**a**), surface morphology of silk sheet after cocoon softening using cocoonase enzyme (**b**), and surface morphology of silk sheet after treatment with Na_2_CO_3_ (**c**)

It was also very much pertinent to know the changes in structural features of silk sheets that are being treated with buffer, enzyme, and chemical. SEM result analysis presented vibrant variation in silk cocoon sheet fiber surface when cocoon softening was done using buffer, cocoonase enzyme, and Na_2_CO_3_ chemical. Silk cocoon sheet obtained after treating/cooking with buffer only (Fig. 16a) was compared with the silk cocoon sheet treated with cocoonase enzyme and Na_2_CO_3_ chemical. Result revealed that silk cocoon sheet treated with cocoonase enzyme holds the natural color of tasar silk (Fig. 16b), smoothness, and luster compared with the cocoon sheet treated with Na_2_CO_3_ chemical (Fig. 16c).

**Fig. 17:**
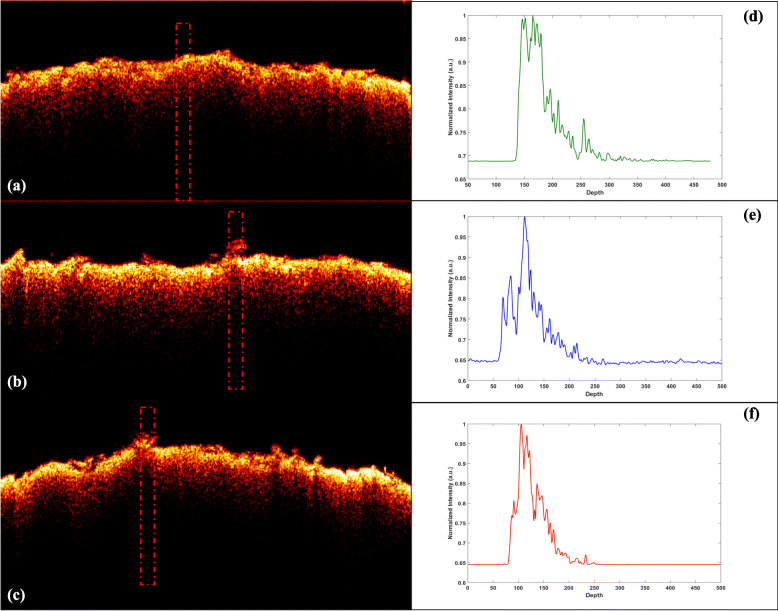
Acquired OCT B-scans of different treatment of silk cocoon sheet with their corresponding A-line. OCT B-scan of only buffer treated (control) silk sheet (**a**); B-scan of silk cocoon sheet treated with cocoonase (**b**); B-scan of silk cocoon sheet treated with Na_2_CO_3_ chemical (**c**). Image size 6 mm (width) × 0.9 mm (height). Red dotted rectangular boxes show the “ROI” region and presented in middle panel. **d**, **e**, **f** Averaged A-scans of “ROIs” for control, treatment with cocoonase enzyme, and with Na_2_CO_3_ chemical, respectively

It might be further interesting to know the micro-structural changes in silk sheets subjected to chemical- and enzyme-based treatment. To compare and analyze the treatment effect with only buffer, cocoonase enzyme, and chemical at different stages, normalized depth profiles were plotted. Treatment effect was mainly studied using optical coherence tomography (OCT) image analysis while comparison of morphology was performed using histological images obtained with the treatment of silk cocoon sheet with buffer only, cocoonase enzyme, and Na_2_CO_3_ chemical. OCT B-scan image of silk cocoon sheet kept under control (Fig. 17a), cocoonase-treated cocoon sheet (Fig. 17b), and Na_2_CO_3_ chemical-treated sheet (Fig. 17c) were obtained where the zoomed red dotted rectangle box showed the region of interest (ROI). Figure 17 d represents A-scan image that gives simplicity of different internal layers in the form of peaks. A-scan image indicated the depth attenuation profile peak of the silk sheet kept under control. The thickness of the silk sheet in Fig. 17d was less in control condition as compared to the treated conditions. The different peaks of the A-scan image represented different layers of the silk cocoon sheet. A-scan profile in Fig. 17e showed the penetration depth of the silk sheet that has been increased, and the thickness has also been increased due to the cocoonase treatment when compared with the control treatment. Treatment of cocoonase also led to the higher number of peaks with higher contrast in A-scan image. A-scan profile in Fig. 17f showed the increased thickness as well as higher contrast.

## Discussion

Resemblances and variations among associated biological sequences obtained by sequence alignment are embodied in the form of phylogenetic trees. A phylogenetic tree or phylogeny was an illustration that depicts the lines of evolutionary descent of different species, organisms, or genes from a common ancestor [7]. In the present study phylogenetic analysis of cocoonase and sericin proteins revealed that these sequences were evolutionarily more conserved in *B. mori* as compared to cocoonase and sericin sequences of other species (Fig. 1 a and b). Secondary structure information of a protein was very much important for folding of a protein into its stable three-dimensional structure or tertiary structure. And predicting protein secondary structures from its sequences has been considered as an intermediate stage bridging the gap between the primary sequences and tertiary structure prediction [81]. Predicted secondary structures (α helix, β turn, extended strand, and random coil) of cocoonase and sericin have been enlisted (Fig. 2 a and b and Supplementary Figures 1a & b). Accurate 8-state secondary structure prediction can significantly give more precise and high resolution on structure-based property analysis. And a valuable method for accurate prediction of 8-state protein secondary structures by a novel deep learning architecture has also been described [81]. Phylogenetic analysis revealed that *B. mandarina* and *B. mori* cocoonase mRNA sequences were closely related, while *A. pernyi* cocoonase mRNA sequence showed little variation [43].

The I-TASSER server was an online workbench for high-resolution modeling of protein structure and function [63]. I-TASSER-based 3D structure prediction of cocoonase and sericin have shown α-helix (red color), 3_10_ helix (blue color), beta sheets (yellow color), turns (cyan color), and coil (purple color) (Fig. 3 a and b). Predicted model having C-score > − 1.5 indicated that these models were of correct global topology. Functional characterization of *A. pernyi* cocoonase protein by predicting its 3D structure using I-TASSER has been reported [43]. ResQ was a model quality assessment program for the local structure quality estimation and used to assess the accuracy of structure models generated by both I-TASSER and other structure prediction method [74]. ResQ-based first model prediction by I-TASSER for cocoonase and sericin revealed that majority of residues in the model were displayed accurately (Fig. 4 a and b). To find the structurally similar analogs of the query proteins [75], TM-align-based identification of the first I-TASSER model was performed against the PDB library [80]. The top 10 PDB proteins that were structurally close to the cocoonase (1eaxA, 1fiwA, 1fizA, 3w94A, 2f91A, 1ekbB, 1bmaA, 1bruP, 1z8gA, 2anyA) and sericin (5n8pA, 5gr8A, 5hyxB, 5gijB, 2a0zA, 4ecnA, 3cigA, 4mn8A, 6gffI, 4lxrA) proteins have been shown in Fig. 5 a and b. The structural alignments between the query and the 10 closest proteins have been ranked based on the TM-score [79].

COFACTOR has been used to predict the protein function by using 3D structural information of proteins [62, 77]. Also, COFACTOR, a protein function prediction webserver, predicts EC numbers and ligand-binding sites by using structural properties of proteins [62]. Varying ligand-binding site residues as well as functional template for cocoonase and sericin with a high confidence scores (C-score = 0.95 and C-score = 0.09, respectively) have been predicted, where high C-score (0–1) indicated that deduced structures (Fig. 6 a and b) were stable. COFACTOR-based protein function prediction by finding Enzyme Commission number (EC) and ligand-binding sites has also been described [77]. For cocoonase, a template PDB ID:1z8gA with EC number 3.4.21.106 (a hepsin belonging to peptidase family S1A), active-site residues (45, 88, 180, 182, 183, and 197), and C-score of 0.75 have been predicted (Fig. 7a). Similarly, for sericin, another template PDB ID: 3h09B with EC number 3.4.21.72 (a IgA-specific serine endopeptidase belonging to peptidase family S1A), active-site residues (74 and 130), and C-score 0.153 have been predicted (Fig. 7b). Cscore^EC^ was the confidence score for the EC number prediction, and its values range in between 0 and 1, where a higher score indicated a more reliable EC number prediction [62]. DEEPre sequence-based enzyme EC number prediction by deep learning method having ability to capture the functional difference of enzyme isoforms has also been described [44].

B-factor was a value that indicated the extent of the inherent thermal mobility of residues/atoms in proteins [75]. Predicted normalized B-factors of cocoonase for the helix and strand regions were negative or close to zero (Fig. 8a) indicating that these regions were structurally more stable. On the other hand predicted normalized B-factors of sericin for the helix and strand regions were close to zero (Fig. 8b) suggesting that these regions were structurally less stable (Fig. 8b). Aldose reductase and its analogs have a good experimental set of structures to explore the importance of B-factor-based analysis [5]. The Ramachandran plot was an important tool used in the analysis of protein structures [27]. Ramachandran plots have been used to validate protein three-dimensional structures determined using crystallographic methods, NMR spectroscopy, or even computational modeling techniques [11] Also, ϕ and ψ torsion angles in a blocked monopeptide have played a central role in understanding protein structure [60]. A Ramachandran plot out using PROCHECK for cocoonase (Fig. 9a) and sericin (Fig. 9b) confirmed the good quality of model. The six graphs on the main chain parameters of cocoonase (Fig. 10a) and sericin (Fig. 10b) plots indicated the structure (represented by solid square) compared with well-refined structures at a similar resolution. Similarly, predictive study on six graphs on main chain parameters (namely Ramachandran plot quality, peptide bond planarity, inappropriate non-bonded interactions, C alpha tetrahedral distortion, and main-chain hydrogen bond energy for HIV-1 Virion Infectivity Factor (vif)) has been carried out [4]. In stereochemical quality of protein structures in some cases, the trend was dependent on the resolution while in other cases it remained independent of it [41].

UCSF Chimera has been used for the prediction of the hydrophobic and hydrophilic regions of the protein, single atom, and hydrogen bond in the protein models of cocoonase and sericin shown by I-TASSER (Fig. 11a–f). An UCSF Chimera tool, RRDistMaps, has been developed to compute the generalized maps in order to analyze pairwise variations in intramolecular contacts. RRDistMaps has an interactive utility to visualize conformational changes, both local (binding-site residues) and global (hinge motion), between unbound and bound proteins through distance patterns [13]. Another web application and a downloadable tool, ConEVA, has been developed that was useful for a range of contact-related analysis and evaluations including predicted contact comparison, investigation of individual protein folding using predicted contacts, and analysis of contacts in varying structures [1].

Identification of domains in protein sequences was a key step towards structural and functional annotation of protein [50]. Domains were allied with structures, and their identification has been used to infer the protein structure [46, 70]. Prediction of domain might also be helpful in various analysis like comparative analysis of domain families [76], evolution of protein and domain structure and function [20, 55], prediction of protein-protein interactions [15, 24, 35] as well as in identifying the evolutionary relationships of multidomain proteins [65]. A trypsin-like serine protease as a conserved domain in *B. mori* cocoonase (Fig. 12a), while no conserved domain for sericin protein (Fig. 12b) has been predicted, indicates that cocoonase has proteolytic activity. In *A. pernyi* cocoonase, mRNA sequence common conserved region of trypsin-like serine protease and peptidase S1 domain has been predicted [43]. The co-expression scores in STRING v10 have been computed using a revised and improved pipeline [66], making use of all microarray gene expression experiments deposited in NCBI Gene Expression Omnibus, NCBI GEO [6]. In the present study, an interacting network of cocoonase (Fig. 13a) and sericin (Fig. 13b) proteins has been found indicating that these proteins have significant interaction. The color saturation of the edges denotes the confidence score of a functional association. Also, protein–protein interaction network of *Litopenaeus vannamei* haemocytes has also been reported [26]. STRING-based HSP70 protein interacting network analysis revealed that HSP90AA1, HSF1, HSP90AB1, DNAJB1, DNAJB6, BAG3, LOC783577, DNAJC7, BAG1, and DNAJC2 proteins were found to be interacting with HSP70 [64].

Identification of stage at which maximum collection of cocoonase might be achieved was significantly important. Therefore, proper stage selection followed by collection of cocoonase from pupa using drop-by-drop method has been performed (Fig. 14 a and b). Cocoon degumming by chemical treatment resulted in deterioration in silk quality and tensile strength of silk and release of relatively more sericin (Table 1). However, cocoonase enzyme-based degumming might have advantages over chemical-based degumming of the cocoon. Degumming effect of various chemicals on the silk yarn of Chinese, Bangalore, and Murshidabad has been studied showing maximum degumming effect with ethylene diamine as compared to Marseille soap, Na_2_CO_3_, tartaric acid, and alcalase enzyme [14]. Silk degumming and sericin extraction have also been investigated by using 2% anhydrous sodium carbonate where degumming loss percentage and the recovery rate of sericin were 26.1% and 75.5%, respectively [68]. Soap-soda-based degumming effect on weight loss, absorbency, bending length, breaking load, elongation at break, and crease recovery using mulberry, muga, tasar, and ericream silk substrates revealed that muga, tasar, and ericraem silks required more time and severe conditions for sericin removal as compared to mulberry silk, indicating that sericin was more strongly embedded in wild silk as compared to mulberry silk [67]. Silk degumming and sericin extraction from silk fibers has also been studied using enzymatic methods, high temperature, and high-pressure methods to compare the fiber whiteness, brightness, weight loss, breaking strength, and elongation. Result revealed that enzymatic process with 8% savinase and 1100 °C of high temperature was comparable signifying that this might also be used as an alternative method for sericin degumming [2]. SDS-PAGE separation of purified *A. mylitta* native cocoonase showed its molecular weight of 25–26 kDa (Fig. 15).

Earlier scanning electron microscopy (SEM) and mechanical testing-based analysis revealed that the silk sheet of *Antherina suraka* cocoon was less compact, with greater thickness and lower tensile strength and stiffness than that of *B. mori* [57]. SEM-based surface morphology of tasar silk fiber waste protein (sericin) has also been carried out [29]. Recently, a SEM-based study revealed that the silk fibers obtained from peptide-treated silkworm were smooth in texture and at least two times thicker than untreated counterparts [30]. SEM analysis of silk cocoon sheets treated with buffer only (Fig. 16a), cocoonase enzyme (Fig. 16b), and Na_2_CO_3_ chemical (Fig. 16c) showed noticeable variations, where cocoonase enzyme-treated silk cocoon sheet clutches the natural color of tasar silk, smoothness, and luster compared with the cocoon sheet treated with Na_2_CO_3_ chemical. Effect of cocoonase on degumming of silk cocoon, elemental analysis, and MALDI-TOF-TOF analysis of cocoonase has been carried out showing the similar degumming effect of cocoonase on silk sheet [53].

Optical microscopy-based analysis of cocoon sheet has also been performed to detect micro-structural correlation between OCT B-scan and cocoon sheet cross-section. Present study revealed morphological variations under control, cocoon sheet degummed with cocoonase enzyme in which sericin is released showing treatment variations observed in the OCT B images (Fig. 17a–c). Sericin a glue protein that binds with the fibroin protein and its removal were the target of chemical and enzyme-based treatment/degumming that resulted in fibroin (silk thread) for easy reeling. Observable variations in the peaks of A-scan images (Fig. 17d–f) indicating release of sericin by chemical and enzyme treatment have been recorded. Degumming with Na_2_CO_3_ affected the thermal stability and mechanical properties of silk fibroin membranes, and Na_2_CO_3_ degumming process caused serious damage to the heavy chain of silk fibroin [69]. OCT has been used in getting the 2D images of sub-surface structures in wheat-infected leaf [58], OCT-based structural changes in rice leaves during senescence [3], and morphological characterization of rice leaf bulliform and aerenchyma regions [37].

## Conclusion

Phylogenetic analysis of cocoonase and sericin revealed their evolutionary relationship between different species. Secondary structure as well as 3D structure prediction of cocoonase and sericin disclosed the atomic structure while I-TASSER predicted the most stable structure. Both proteins were searched in PDB for predicting their structural closeness to the target in the PDB, ligand-binding sites, and active sites. EC predictions revealed that cocoonase (a hepsin and belongs to peptidase family S1A) has EC number 3.4.21.106, while sericin (a IgA-specific serine endopeptidase that also belongs to peptidase family S1A) holds EC number 3.4.21.72. Stability analysis by normalized B-factor and Ramachandran plot showed that cocoonase contains residues in the most favored region. UCSF Chimera showed the hydrophobicity nature of both proteins representing the presence of hydrogen bond between the atoms of protein. Natural cocoonase treatment-induced weight loss presented its degumming activity. Purification and subsequent SDS-PAGE analysis of cocoonase showed its molecular weight of 25–26 kDa. Silk cocoon sheet obtained after the degumming with cocoonase exhibited the natural color, smoothness, and luster, while silk sheet treated with chemicals showed comparable weaker strength. SEM-and OCT-based study also evidenced morphological variations in control, cocoonase, and chemical degummed silk cocoon sheet.

## Supplementary Information

**Additional file 1: Supplementary Figure 1.** Predicted secondary structure of cocoonase (a) and sericin (b). In cocoonase and sericin lines in different colors represent different secondary structures: Blue for α helix, green for β turn, red for extended strand, and purple for random coil.

## Data Availability

Materials used and data generated are available.

## References

[1] Adhikari B, Nowotny J, Bhattacharya D, Hou J, Chen J (2016). ConEVA: a toolbox for comprehensive assessment of protein contacts. BMC Bioinformatics.

[2] Anis P, Capar G, Toprak T, Yener E (2016). Sericin removal from silk fibers with eco-friendly alternative methods. Tekstil ve Konfeksiyon.

[3] Anna T, Chakraborty S, Cheng C-Y, Srivastava V, Chiou A, Kuo W-C (2019). Elucidation of microstructural changes in leaves during senescence using spectral domain optical coherence tomography. Sci Rep.

[4] Balaji S, Kalpana R, Shapshak P (2006). Paradigm development: comparative and predictive 3D modeling of HIV-1 Virion Infectivity Factor (Vif). Bioinformation.

[5] Balendiran GK, Pandian JR, Drake E, Vinayak A, Verma M, Cascio D (2014). B-factor analysis and conformational rearrangement of aldose reductase. Curr Proteomics.

[6] Barrett T, Wilhite SE, Ledoux P, Evangelista C, Kim IF, Tomashevsky M, Marshall KA, Phillippy KH, Sherman PM, Holko M, Yefanov A, Lee H, Zhang N, Robertson CL, Serova N, Davis S, Soboleva A (2013). NCBI GEO: archive for functional genomics data sets–update. Nucleic Acids Res.

[7] Baum D (2008). Reading a phylogenetic tree: the meaning of monophyletic groups. Nat Educ.

[8] Berger E, Kafatos FC, Felsted RL (1971). Cocoonase III purification, preliminary characterization, and activation of the zymogen of an insect protease. J Biol Chem.

[9] Borah MP, Bulbul B (2009). Enzymatic treatment on cooking and reeling of muga silk (*Aniheraea assama*) cocoon. Asian J Home Sci.

[10] Bradford MM (1976). A rapid and sensitive method for quantitation of microgram quantities of protein utilizing the principle of protein-dye binding. Anal Biochem.

[11] Carugo O, Carugo KD (2013). Half a century of Ramachandran plots. Acta Crystallogr.

[12] Chakraborty S, Muthulakshmi M, Vardhini D, Jayaprakash P, Nagaraju J, Arunkumar KP (2015). Genetic analysis of Indian tasar silkmoth (*Antheraea mylitta*) populations. Sci Rep.

[13] Chen JE, Huang CC, Ferrin TE (2015). RRDistMaps: a UCSF Chimera tool for viewing and comparing protein distance maps. Bioinformatics.

[14] Chopra S, Gulrajani ML (1994). Comparative evaluation of various methods of degumming silk. Indian J Fibre Textile Res.

[15] Deng M, Mehta S, Sun F, Chen T (2002). Inferring domain-domain interactions from protein-protein interactions. Genome Res.

[16] Deo AS, Prasad S, Ram MK, Sinha AK, Pandey JP, Pandey DM (2018). A computational approach to study the ESTs, miRNA and SNPs in *Antheraea mylitta*. Intern J Com Bioinfo In Silico Model.

[17] Devi R (2012). Biotechnological application of proteolytic enzymes in post cocoon technology. Intern J Sci Nat.

[18] Devi YR, Singh LR, Devi SK (2012). Comparative evaluation of commonly adopted methods of oak tasar silk cocoon cooking. Intern J Curr Res Review.

[19] Felsted RL, Kramer KJ, Law JH, Beroer E, Kafatos FC (1973). Cocoonase IV. Mechanism of activation of prococoonase from *Antheraea polyphemus*. J Biol Chem.

[20] Fong JH, Geer LY, Panchenko AR, Bryant SH (2007). Modeling the evolution of protein domain architectures using maximum parsimony. J Mol Biol.

[21] Franceschini A, Szklarczyk D, Frankild S, Kuhn M, Simonovic M, Roth A, Lin J, Minguez P, Bork P, von Mering C, Jensen LJ (2013). STRING v9.1: protein-protein interaction networks, with increased coverage and integration. Nucleic Acids Res.

[22] Fukumori H, Teshiba S, Shigeoka Y, Yamamoto K, Banno Y, Aso Y (2014). Purification and characterization of cocoonase from the silkworm *Bombyx mori*. Biosci Biotechnol Biochem.

[23] Geoujon C, Deleage G (1995). SOPMA: significant improvements in secondary structure prediction from multiple alignments. Comput Appl Biosci.

[24] Guimarães K, Jothi R, Zotenko E, Przytycka T (2006). Predicting domain-domain interactions using a parsimony approach. Genome Biol.

[25] Gulrajani ML (1992). Degumming of silk. Coloration Related Topics.

[26] Hao T, Zhao L, Wu D, Wang B, Feng X, Wang E, Sun J (2019). The protein–protein interaction network of *Litopenaeus vannamei* haemocytes. Front Physiol.

[27] Ho BK, Brasseur R (2005). The Ramachandran plots of glycine and pre-proline. BMC Struct Biol.

[28] Iizuka E (2000). Physical properties of silk thread from cocoons of various wild silkmoths including domestic silk moth. Int J Wild Silkmoth Silk.

[29] Jena K, Pandey JP, Kumari R (2018). Tasar silk fiber waste sericin: new source for anti-elastase, anti-tyrosinase and anti-oxidant compounds. Int J Biol Macromol.

[30] Jha S, Bhattacharyya P, Ghosh A, Gupta PD, Mandal P (2019). Impact of feeding low molecular weight mulberry peptides on cocoon and silk development by *Bombyx mori* L. (Bombycidae). Indian J Sericulture.

[31] Johnny RV, Karpagam S (2012). Degumming of silk using protease enzyme from *Bacillus* species. Intern J Sci Nat.

[32] Jones DT, Taylor WR, Thornton JM (1992). The rapid generation of mutation data matrices from protein sequences. Bioinformatics.

[33] Kabsch W, Sander C (1983). Dictionary of protein secondary structure: pattern recognition of hydrogen-bonded and geometrical features. Biopolymers.

[34] Kafatos FC, Williams CM (1964). Enzymatic mechanism for the escape of certain moths from their cocoons. Science.

[35] Kanaan SP, Huang C, Wuchty S, Chen DZ, Izaguirre JA (2009). Inferring protein-protein interactions from multiple protein domain combinations. Methods Mol Biol.

[36] Kanost MR, Clem RJ (2012) Insect proteases. In “Insect molecular biology and biochemistry” (LI Gilbert ed) Elsevier, New York, pp 346-364.

[37] Kim H, Du X, Kim S, Kim P, Wijesinghe RE, Yun BJ, Kim J (2019). Non-invasive morphological characterization of rice leaf bulliform and aerenchyma cellular regions using low coherence interferometry. Appl Sci.

[38] Kramer KJ, Felsted RL, Law JH (1973). Cocoonase V. Structural studies on an insect serine protease. J Biol Chem.

[39] Kumar S, Stecher G, Li M, Knyaz C, Tamura K (2018). MEGA X: molecular evolutionary genetics analysis across computing platforms. Mol Biol Evol.

[40] Laemmli UK (1970). Cleavage of structural proteins during the assembly of the head of bacteriophage T4. Nature.

[41] Laskowski RA, MacArthur MW, Moss DS, Thornton JM (1993). PROCHECK: a program to check the stereochemical quality of protein structures. J Appl Cryst.

[42] Laskowski RA, Rullmannn JA, MacArthur MW, Kaptein R, Thornton JM (1996). AQUA and PROCHECK-NMR: programs for checking the quality of protein structures solved by NMR. J Biomol NMR.

[43] Lata S, Pandey DM, Pandey JP (2013). Unraveling the sequence similarities, conserve domain and 3D structure of cocoonase to gain insights into their functional integrity. Int J Comput Bioinfo In Silico Model.

[44] Li Y, Wang S, Umarov R, Xie B, Fan M, Li L, Gao X (2018). DEEPre: sequence-based enzyme EC number prediction by deep learning. Bioinformatics..

[45] Mahmoodi NM, Moghimi F, Arami M, Mazaheri M (2010). Silk degumming using microwave irradiation as an environmentally friendly surface modification method. Fibers Polymers.

[46] Marchler-Bauer A, Anderson JB, Chitsaz F, Derbyshire MK, DeWeese-Scott C, Fong JH, Geer LY, Geer RC, Gonzales NR, Gwadz M, He S, Hurwitz DI, Jackson JD, Ke Z, Lanczycki CJ, Liebert CA, Liu C, Lu F, Lu S, Marchler GH, Mullokandov M, Song JS, Tasneem A, Thanki N, Yamashita RA, Zhang D, Zhang N, Bryant SH (2009). CDD: specific functional annotation with the conserved domain database. Nucleic Acids Res.

[47] Marchler-Bauer A, Lu S, Anderson JB, Chitsaz F, Derbyshire MK, DeWeese-Scott C, Fong J, Gee LY, Geer RC, Gonzales NR, Gwadz M, Hurwitz DI, Jackson JD, Ke Z, Lanczycki CJ, Lu F, Marchler GH, Mullokandov M, Omelchenko MV, Robertson CL, Song JS, Thanki N, Yamashita RA, Zhang D, Zhang N, Zheng C, Bryant SH (2011). CDD: a conserved domain database for the functional annotation of proteins. Nucleic Acids Res.

[48] Marchler-Bauer A, Derbyshire MK, Gonzales NR, Lu S, Chitsaz F, Geer LY, Geer RC, He J, Gwadz M, Hurwitz DI, Lanczycki CJ, Lu F, Marchler GH, Song JS, Thanki N, Wang Z, Yamashita RA, Zhang D, Zheng C, Bryant SH (2015). CDD: NCBI’s conserved domain database. Nucleic Acids Res.

[49] Meng EC, Pettersen EF, Couch GS, Huang CC, Ferrin TE (2006). Tools for integrated sequence-structure analysis with UCSF Chimera. BMC Bioinfo.

[50] Ochoa A, Llinás M, Singh M (2011). Using context to improve protein domain identification. BMC Bioinformatics.

[51] Pandey DM, Pandey JP (2014). Cocoonase enzyme: current and future perspectives. Austin J Biotechnol Bioeng.

[52] Pandey JP, Mishra PK, Kumar D, Sinha AK, Prasad BC, Singh BMK, Paul TK (2011). Possible efficacy of 26 kDa *Antheraea mylitta* cocoonase in cocoon cooking. Int J Biol Chem.

[53] Pandey JP, Sinha AK, Jena K, Gupta VP, Kundu P, Pandey DM (2018). Prospective utilization of *Antheraea mylitta* cocoonase and its molecular harmony with nature. Int J Adv Res.

[54] Pettersen EF, Goddard TD, Huang CC, Couch GS, Greenblatt DM, Meng EC, Ferrin TE (2004). UCSF Chimera—a visualization system for exploratory research and analysis. J Comput Chem.

[55] Przytycka T, Davis G, Song N, Durand D (2006). Graph theoretical insights into Dollo parsimony and evolution of multidomain proteins. J Comput Biol.

[56] Rajkhowa R (2000). Structure property correlation of non-mulberry and mulberry silk fibres. Int J Wild Silkmoth Silk.

[57] Randrianandrasana M, Wu W-Y, Carney DA, Johnson AJW, Berenbaum MR (2017). Structural and mechanical properties of cocoons of *Antherina suraka* (Saturniidae, Lepidoptera), an endemic species used for silk production in Madagascar. J Insect Sci.

[58] Rateria A, Mohan M, Mukhopadhyay K, Poddar R (2019). Investigation of *Puccinia triticina* contagion on wheat leaves using swept source optical coherence tomography. Optik.

[59] Renuka G, Shamitha G (2015). Studies on the biodiversity of tasar ecoraces *Antheraea mylitta* Drury. J Entomol Zoology Studies.

[60] Rose GD (2019). Ramachandran maps for side chains in globular proteins. Proteins.

[61] Roy A, Kucukural A, Zhang Y (2010). I-TASSER: a unified platform for automated protein structure and function prediction. Nat Protoc.

[62] Roy A, Yang J, Zhang Y (2012). COFACTOR: an accurate comparative algorithm for structure-based protein function annotation. Nucleic Acids Res.

[63] Roy A, Xu D, Poisson J, Zhang Y (2011). A protocol for computer-based protein structure and function prediction. J Vis Exp.

[64] Singh R, Gurao A, Rajesh C, Mishra SK, Rani S, Behl A, Kumar V, Kataria RS (2019). Comparative modeling and mutual docking of structurally uncharacterized heat shock protein 70 and heat shock factor-1 proteins in water buffalo. Veterinary World.

[65] Song N, Joseph JM, Davis GB, Durand D (2008). Sequence similarity network reveals common ancestry of multidomain proteins. PLoS Comput Biol.

[66] Szklarczyk D, Franceschini A, Wyder S, Forslund K, Heller D, Huerta-Cepas J, Simonovic M, Roth A, Santos A, Tsafou KP, Kuhn M, Bork P, Jensen LJ, von Mering C (2015). STRING v10: protein-protein interaction networks, integrated over the tree of life. Nucleic Acids Res.

[67] Teli MD, Rane VM (2011). Comparative study of the degumming of mulberry, muga, tasar and ericream Silk. Fibres Textiles Europe.

[68] Wang H, Guo Y (2011). Technical process of silk degumming and sericin extracting. Adv Mat Res.

[69] Wang Z, Yang H, Li W, Li C (2019). Effect of silk degumming on the structure and properties of silk fibroin. J Textile Inst.

[70] Wilson D, Pethica R, Zhou Y, Talbot C, Vogel C, Madera M, Chothia C, Gough J (2009). SUPERFAMILY--sophisticated comparative genomics, data mining, visualization and phylogeny. Nucleic Acids Res.

[71] Yang J, Roy A, Zhang Y (2013). Protein-ligand binding site recognition using complementary binding-specific substructure comparison and sequence profile alignment. Bioinformatics.

[72] Yang J, Roy A, Zhang Y (2013). BioLiP: a semi-manually curated database for biologically relevant ligand-protein interactions. Nucleic Acids Res.

[73] Yang J, Wang Y, Zhang Y (2016). ResQ: an approach to unified estimation of B-factor and residue-specific error in protein structure prediction. J Mol Biol.

[74] Yang J, Yan R, Roy A, Xu D, Poisson J, Zhang Y (2015). The I-TASSER suite: protein structure and function prediction. Nat Methods.

[75] Yang J, Zhang Y (2015). I-TASSER server: new development for protein structure and function predictions. Nucleic Acids Res.

[76] Ye Y, Godzik A (2004). Comparative analysis of protein domain organization. Genome Res.

[77] Zhang C, Freddolino PL, Zhang Y (2017). COFACTOR: improved protein function prediction by combining structure, sequence and protein-protein interaction information. Nucleic Acids Res.

[78] Zhang Y (2008). I-TASSER server for protein 3D structure prediction. BMC Bioinformatics.

[79] Zhang Y, Skolnick J (2004). Scoring function for automated assessment of protein structure template quality. Proteins.

[80] Zhang Y, Skolnick J (2005). TM-align: a protein structure alignment algorithm based on the TM-score. Nucleic Acids Res.

[81] Zhang B, Li J, Lü Q (2018). Prediction of 8-state protein secondary structures by a novel deep learning architecture. BMC Bioinformatics.

