# Supplementary figures and images for: Study on cocoonase, sericin, and degumming of silk cocoon: computational and experimental
Source: J Genet Eng Biotechnol. 2021 Feb 16;19:32. doi: 10.1186/s43141-021-00125-2 (PMC7886927; doi:10.1186/s43141-021-00125-2)

**Supplementary Figure-1**


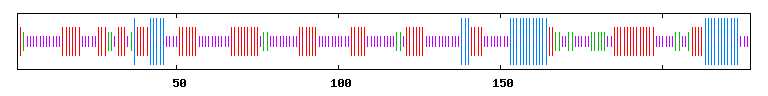

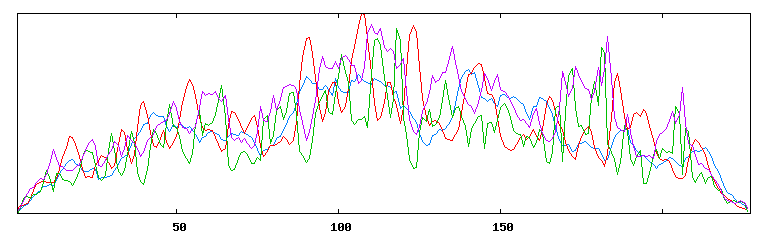


**(a)**


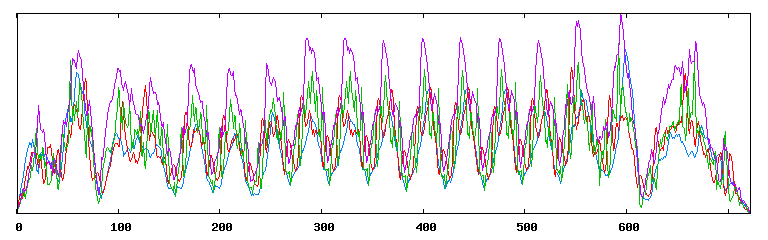

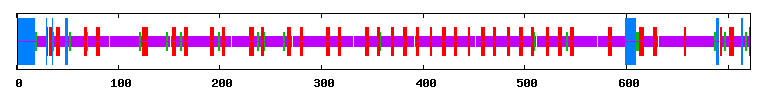


**(b)**

Supplement: Supplementary file 1 — Additional file 1: Supplementary Figure 1. Predicted secondary structure of cocoonase (a) and sericin (b). In cocoonase and sericin lines in different colors represent different secondary structures: Blue for α helix, green for β turn, red for extended strand, and purple for random coil. [file 43141_2021_125_MOESM1_ESM.docx]
